# BAM15 treats mouse sepsis and kidney injury, linking mortality, mitochondrial DNA, tubule damage, and neutrophils

**DOI:** 10.1172/JCI152401

**Published:** 2023-04-03

**Authors:** Naoko Tsuji, Takayuki Tsuji, Tetsushi Yamashita, Naoki Hayase, Xuzhen Hu, Peter S.T. Yuen, Robert A. Star

**Affiliations:** Renal Diagnostics and Therapeutics Unit, National Institute of Diabetes and Digestive and Kidney Diseases (NIDDK), NIH, Bethesda, Maryland, USA.

**Keywords:** Nephrology, Innate immunity, Mitochondria

## Abstract

Sepsis pathogenesis is complex and heterogeneous; hence, a precision-medicine strategy is needed. Acute kidney injury (AKI) following sepsis portends higher mortality. Overproduction of mitochondrial ROS (mtROS) is a potential mediator of sepsis and sepsis-induced AKI. BAM15, a chemical uncoupler, dissipates mitochondrial proton gradients without generating mtROS. We injected BAM15 into mice at 0, 6, or 12 hours after cecal ligation and puncture (CLP), and these mice were treated with fluids and antibiotics. BAM15 reduced mortality, even after 12 hours, when mice were ill, and BAM15 reduced kidney damage and splenic apoptosis. Serial plasma and urinary mitochondrial DNA (mtDNA) levels increased after CLP and decreased after BAM15 administration (at 0 or 6 hours). In vitro septic serum proportionately increased mtROS overproduction and mtDNA release from kidney tubule cells, which BAM15 prevented. BAM15 decreased neutrophil apoptosis and mtDNA release; neutrophil depletion counteracted BAM15 benefits. Further, mtDNA injection in vivo replicated inflammation and kidney injury, which was prevented by BAM15. A large dose of exogenous mtDNA reversed protection by BAM15. We conclude that BAM15 is an effective preventive and therapeutic candidate in experimental sepsis and that BAM15 and mtDNA, a potential drug-companion diagnostic/drug-efficacy pair for clinical sepsis, are mechanistically linked via mtROS.

## Graphical abstract

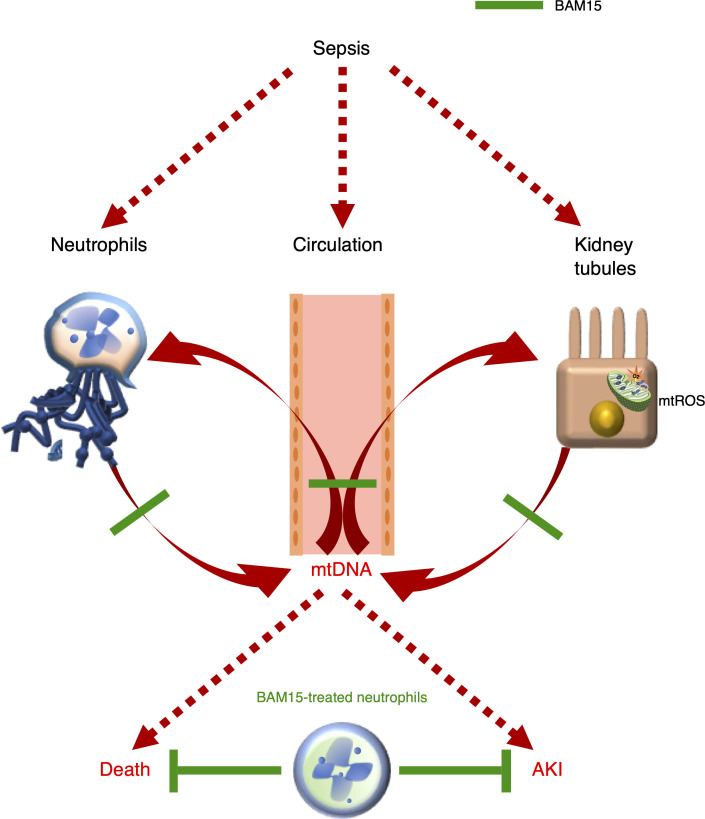

## Introduction

Sepsis is caused by severe infection and progresses to multiple organ failure, including acute kidney injury (AKI). Globally, 48.9 million sepsis cases and 11 million sepsis-related deaths were reported, representing 20% of all global deaths annually (1). One-third of patients with sepsis develop AKI (sepsis-AKI), with a considerable increase in mortality (2). Sepsis accounts for 40% of AKI among critically ill patients (3), and sepsis-AKI has higher mortality than other forms of AKI (1). Even with infectious source control, antibiotics, and renal replacement therapy (RRT), sepsis-induced AKI portends a very high mortality. No effective drugs can prevent or treat human sepsis or sepsis-induced AKI, in part because of multifactorial pathophysiologies that differ among patients with clinical sepsis. Clinical trials are difficult to perform because it is unclear whom to treat, what drug to use, and how to dose. A targeted precision medicine approach has been suggested (4, 5), which would be enhanced by codevelopment of drugs and paired biomarkers, as in cancer (6).

Mitochondrial DNA (mtDNA), recognized recently as a damage-associated molecular pattern (DAMP), is released from injured tissues and circulates and activates immune cells (7). We and others have shown that cell-free mtDNA (cfmtDNA) is released into the circulation during the early phase of sepsis (8, 9) and also in patients with severe SARS-CoV-2 infection (10-12). In sepsis, mitochondrial debris, including mtDNA, contributes to AKI, with overproduction of renal mitochondrial superoxide via TLR9, while DNase treatment diminishes both AKI and renal mitochondrial superoxide (8). Thus, cfmtDNA might be both a biomarker and a therapeutic target of disease severity in sepsis-AKI. However, cfmtDNA has not been evaluated as a therapeutic efficacy biomarker in sepsis or sepsis-AKI.

BAM15 ([2-fluorophenyl]-{5-oxadiazolo(3,4-e]pyrazin-5-yl)}amine) is a mitochondrial uncoupler that can protect the kidney from cold storage–induced damage (13) and ischemic injury (14). Mitochondrial uncouplers transport protons from the mitochondrial inner membrane space into the mitochondrial matrix independently of ATP synthase and thus uncouple nutrient metabolism from ATP generation. Mitochondrial uncouplers paradoxically decrease superoxide production by decreasing electron dwell time and increasing the speed of electron transfer through the electron transport chain (ETC) (15–17). BAM15 has less cytotoxicity and fewer off-target effects on plasma membrane depolarization compared with other uncouplers (14). The therapeutic potential of mitochondrial uncouplers has been investigated for treating obesity (18), type 2 diabetes (19), and ischemic reperfusion injury–induced AKI (14), but not in sepsis or sepsis-AKI. In endotoxin- or cisplatin-induced AKI, mitochondrial and renal function can be restored if mitochondrial damage is prevented by decreasing excess mitochondrial ROS (mtROS) and increasing mitochondrial biogenesis signaling, which is inhibited during AKI (20, 21). Therefore, we investigated the effect of BAM15 as a mitochondria-targeted drug for sepsis-AKI and evaluated mtDNA as an efficacy biomarker for BAM15 treatment.

## Results

### BAM15 increased sepsis survival and reduced sepsis-AKI, even with delayed treatment.

**Figure 1:**
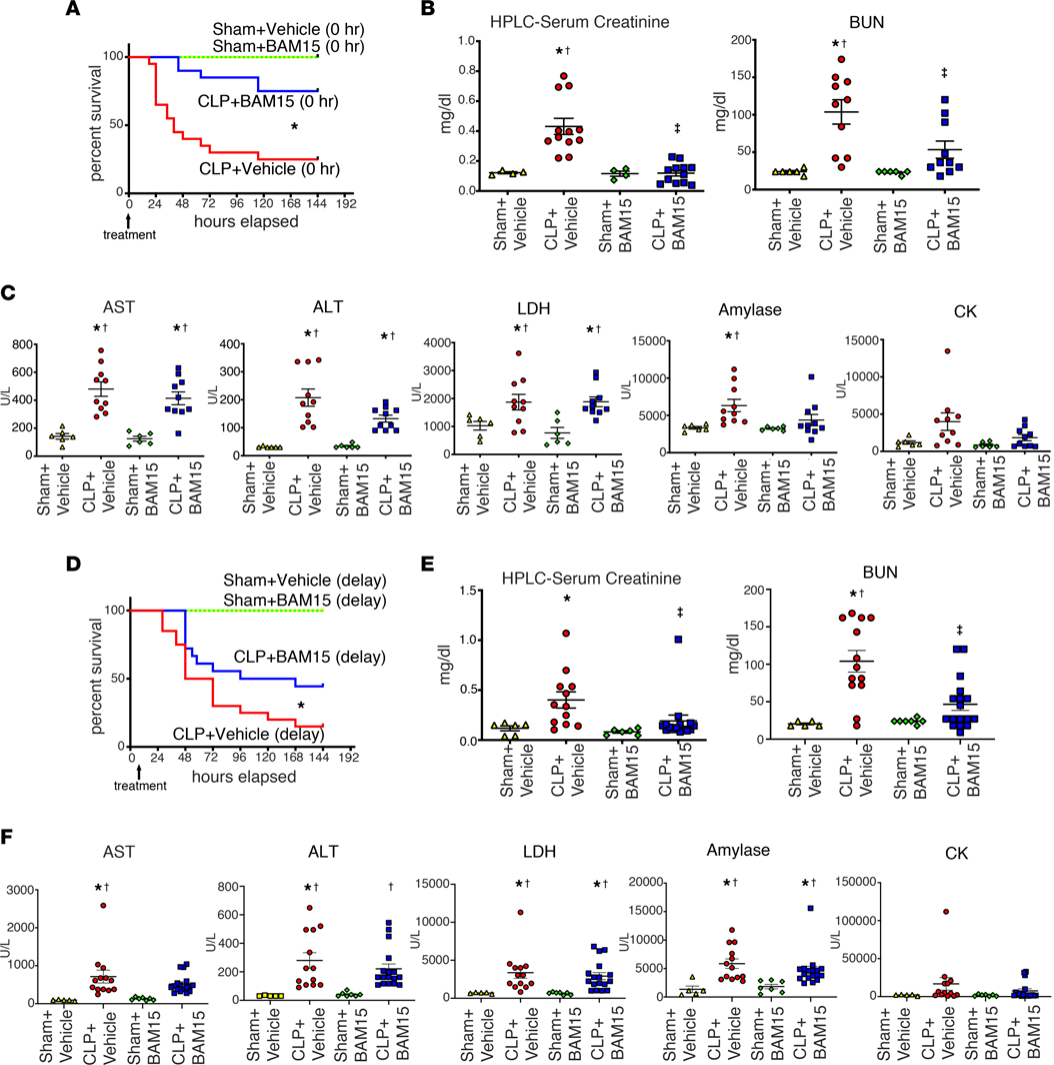
BAM15 treatment improves mortality and AKI in septic mice, even with delayed administration. (**A**) Kaplan-Meier curves of male mice subjected to sham or CLP surgery treated with vehicle (at 0 hours) and mice subjected to sham or CLP surgery treated with BAM15 (5 mg/kg, at 0 hours) for 7 days. Sham+vehicle/BAM15, *n* = 4 each; CLP+vehicle/BAM15, *n* = 20 each. log-rank test. *P < 0.05, CLP+vehicle versus other groups. (**B** and **C**) Serum creatinine measured by HPLC. BUN (**B**), aspartate transferase (AST), alanine transaminase (ALT), lactate dehydrogenase (LDH), amylase, and creatinine kinase (CK) (**C**) by biochemical examination at 18 hours after mice were subjected to sham or CLP surgery and treated with vehicle (at 0 hours) or BAM15 (5 mg/kg, at 0 hours). Data are represented as mean ± SEM of each group (sham+vehicle, *n* = 4–6, CLP+vehicle, *n* = 10–12; sham+BAM15, *n* = 4–6; CLP+BAM15, *n* = 10–12). Šidák’s multiple-comparison test following 1-way ANOVA. *Versus sham+vehicle, *P* < 0.05; ^†^versus sham+BAM15, *P* < 0.05; ^‡^versus CLP+vehicle, *P* < 0.05. (**D**) Kaplan-Meier curves for 7 days of mice subjected to sham or CLP surgery and treated with vehicle (at 6 hours) or BAM15 (5 mg/kg, at 6 hours). Sham+vehicle/BAM15, *n* = 4 each; CLP+vehicle (*n* = 20), BAM15 (*n* = 19). log-rank test. *CLP+vehicle versus other groups, *P* < 0.05. (**E**–**F**) Serum creatinine measured by HPLC. BUN (**E**) and AST, ALT, LDH, amylase, and CK (**F**) measured by biochemical examination at 18 hours after mice subjected to sham or CLP surgery were treated with vehicle (at 6 hours) or BAM15 (5 mg/kg, at 6 hours). Data are represented as mean ± SEM of each group (sham+vehicle, *n* = 5; CLP+vehicle, *n* = 13; sham+BAM15, *n* = 7; CLP+BAM15, *n* = 17). Dunn’s multiple-comparison test following Kruskal-Wallis test. *Versus sham+vehicle, *P* < 0.05; ^†^versus sham+BAM15, *P* < 0.05; ^‡^versus CLP+vehicle, *P* < 0.05.

We first evaluated the impact of BAM15 on clinically relevant outcomes of sepsis and sepsis-AKI. We performed a survival study using a fluid- and antibiotic-treated cecal ligation and puncture (CLP) model of mouse sepsis (22, 23). Early treatment with BAM15 (5 mg/kg i.p.) at the time of CLP surgery substantially increased survival (survival at 7 days: CLP+vehicle, 25%, versus CLP+BAM, 75%; *P* < 0.05) (Figure 1A). Early treatment with BAM15 also improved kidney dysfunction at 18 hours after CLP (serum creatinine: CLP+vehicle versus CLP+BAM15, 0.43 ± 0.05 versus 0.12 ± 0.02 mg/dl, *P* < 0.05; blood urea nitrogen [BUN]: CLP+vehicle, 103.8 ± 16.2 mg/dl versus CLP+BAM15, 53.4 ± 11.5 mg/dl, *P* < 0.05) without a significant effect on other organ damage indices (Figure 1, B and C). The benefit of BAM15 pretreatment was also observed in female mice (Supplemental Figure 1, A–C; supplemental material available online with this article; https://doi.org/10.1172/JCI152401DS1). Delayed treatment with BAM15 (5 mg/kg, i.p.) after the mice became ill (6 hours, at time of first dose of fluid and antibiotics) also decreased mortality (survival at 7 days: CLP+vehicle, 15% versus CLP+BAM15, 44%; *P* < 0.05) and improved kidney dysfunction at 18 hours after CLP (serum creatinine: CLP+vehicle versus CLP+BAM15, 0.40 ± 0.08 versus 0.19 ± 0.06 mg/dl, *P* < 0.05; BUN: CLP+vehicle, 104.0 ± 14.4 mg/dl versus CLP+BAM15: 46.7 ± 8.2 mg/dl, *P* < 0.05) (Figure 1, D–F). BAM15 improved survival even when administered 12 hours after CLP (survival at 7 days: CLP+vehicle, 10% versus CLP+BAM15, 32%; *P* < 0.05) (Supplemental Figure 2). BAM15 treatment did not have any apparent harmful effects on, nonseptic mice subjected to sham surgery.

### BAM15 improved critical vital signs and reduced kidney injury, hypoxia, and oxidative damage in sepsis-AKI.

We also monitored critical vital signs, including mean blood pressure (mBP), heart rate (HR), and body temperature (BT), in CLP mice and mice subjected to sham surgery who received vehicle or BAM15 (5 mg/kg injected at 0 hours). These 3 physiological parameters decreased after CLP surgery in both vehicle- and BAM15-treated mice compared with mice subjected to sham surgery. However, BAM15 significantly blunted reduction of mBP beginning 14 hours after injection, reduction in HR beginning 9 hours after injection, and reduction in BT beginning 6 hours after injection (Supplemental Figure 3A).

**Figure 2:**
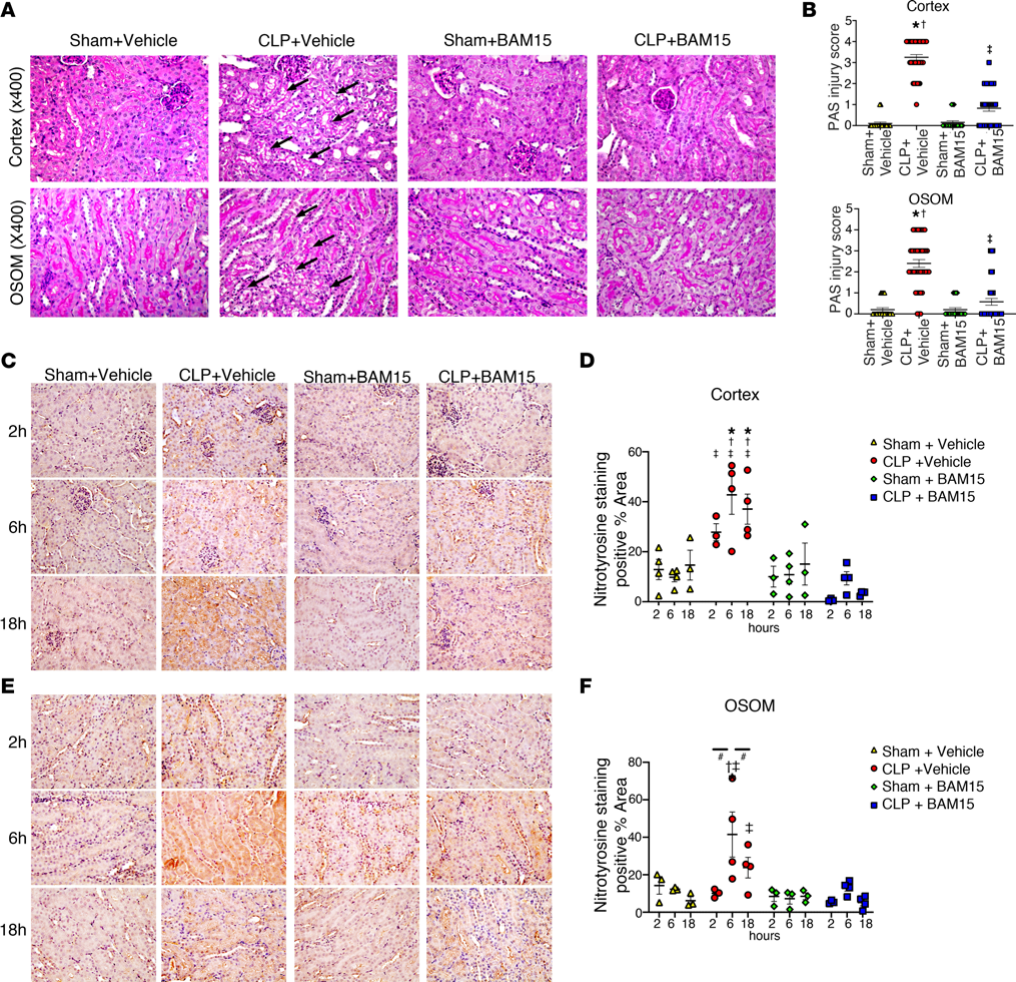
BAM15 reduces injury and oxidative damage via RNS in septic kidney. Periodic acid–Schiff staining of cortex and OSOM in kidneys at 18 hours after mice subjected to sham or CLP surgery were treated with vehicle (at 0 hours) or BAM15 (5 mg/kg, at 0 hours). (**A**) Representative images of cortex and OSOM. Arrows show vacuolization in proximal tubule cells. Original magnification, ×400. (**B**) Tubular damage score in cortex and OSOM of kidney at 18 hours after being subjected to sham (4 mice each, total 20 fields of ×400) or CLP surgery (8 mice each, total 40 fields of ×400) treated with vehicle (at 0 hours) or BAM15 (5 mg/kg, at 0 hours). Data are represented as mean ± SEM. Dunn’s multiple-comparison test following Kruskal-Wallis test. *Versus sham+vehicle, *P* < 0.05; ^†^versus sham+BAM15, *P* < 0.05; ^‡^versus CLP+vehicle, *P* < 0.05. (**A**–**D**) Representative nitrotyrosine images and positive area rate of cortex (**C** and **D**) and OSOM (**E** and **F**) in kidney at 2, 6, and 18 hours after being subjected to sham or CLP surgery and treated with vehicle (at 0 hours) or BAM15 (5 mg/kg, at 0 hours). Original magnification, ×400. The positive area rate was calculated by Fiji/ImageJ (NIH) software. Each circle represents the average of positive area of 3 to 4 fields per mouse kidney (3 to 4 mice/group). Bars show mean ± SEM. Tukey’s multiple-comparison test following 2-way ANOVA test. *Versus sham+vehicle, *P* < 0.05; ^†^versus sham+BAM15, *P* < 0.05; ^‡^versus CLP+vehicle, *P* < 0.05; ^#^comparison between time points, *P* < 0.05.

Kidney tissue damage from sepsis is detected histologically by cytoplasmic vacuoles in tubule cells (23). Cytoplasmic vacuoles were increased in proximal tubule cells of cortex and outer stripe of the outer medulla (OSOM) 18 hours after CLP and were decreased by early treatment with BAM15 (5 mg/kg, i.p.) (Figure 2A). Injury scores were as follows: cortex: CLP+vehicle, 3.3 ± 0.13 versus CLP+BAM15, 0.83 ± 0.14; OSOM: CLP+vehicle, 2.4 ± 0.19 versus CLP+BAM15, 0.58 ± 0.16 (Figure 2B).

Hypoxia is an important contributor to sepsis-induced organ dysfunction. Proximal tubule hypoxia was assessed by pimonidazole incorporation in vivo (24); pimonidazole reacts with cellular proteins at oxygen tensions of less than 10 mmHg. Pimonidazole incorporation was detected in proximal tubule cells in the cortex and the OSOM at 18 hours after CLP and was decreased by early treatment with BAM15 (5 mg/kg i.p.) (hypoxia score: CLP+vehicle, 2.0 ± 0.24 versus CLP+BAM15, 0.75 ± 0.23; OSOM: CLP+vehicle, 2.0 ± 0.24 versus CLP+BAM15, 0.67 ± 0.16, *P* < 0.05) (Supplemental Figure 3, B and C).

We measured kidney reactive nitrogen species (RNS) in mice subjected to CLP with BAM15 to determine the effect of BAM15 on renal oxidants in vivo. Superoxide (O_2_^–^) can react with nitric oxide (NO) to form peroxynitrite (ONOO^–^). Peroxynitrite is a powerful cytotoxic oxidant in septic kidneys (25) that can oxidize thiols and DNA bases or can modify proteins and lipids by nitration. Immunostaining for nitrotyrosine, a product of tyrosine nitration, was performed on kidney sections from mice subjected to CLP and BAM15 administration (at 0 hours) at each time point (2, 6, 18 hours after CLP). Nitrotyrosine was found in tubular epithelial cells from 2 hours (cortex) and 6 hours (OSOM) after mice were subjected to CLP surgery and treated with vehicle. However, BAM15 treatment decreased nitrotyrosine in tubule cells of cortex and OSOM (Figure 2, C–F). Our results confirmed that BAM15 decreased cytotoxic oxidants in septic kidney in vivo. Thus, BAM15 treatment reduced sepsis-induced kidney tissue injury, tissue oxidative damage, and tissue hypoxia.

### BAM15 accelerates mitochondrial respiration in cultured renal tubule cells.

We investigated the direct impact of BAM15 on kidney tubule cell mitochondria (i.e., in the absence of systemic mediators). First, we confirmed that BAM15 acted as a mitochondrial uncoupler in kidney tubule cells by measuring oxygen consumption rate (OCR) and mitochondrial respiratory function in mouse primary cultured proximal tubule cells (mPPTCs) from healthy CD-1 mice. BAM15 (1, 2, 5, 10, 20, 50, and 100 μM) functioned as a mitochondrial uncoupler similar to FCCP (the most commonly used uncoupler). There was a concentration-dependent increase in maximal respiration following addition of 1 μM oligomycin, an ATP synthase inhibitor (Supplemental Figure 4, A–D). Maximal respiration peaked at 20 μM BAM15, which matched the level in response to 5 μM FCCP; 20 μM BAM15 increased maximal respiration more than 20 μM FCCP (*P* < 0.05) (Supplemental Figure 4E).

### BAM15 effects on mitochondrial biogenesis in septic kidney.

We also evaluated the effect of BAM15 on mitochondrial biogenesis after CLP. PGC1α regulates transcription of multiple mitochondrial biogenesis genes and is highly expressed in healthy renal tubules (20). Kidney PGC1α protein expression decreased within 2 hours after CLP and vehicle administration, compared with what occurred in mice subjected to sham surgery and vehicle administration, and PGC1α protein levels did not return to baseline. However, administration of BAM15 prior to CLP increased the expression of PGC1α at 6 hours after CLP, compared with that in mice subjected to CLP and administered with vehicle, and PGC1α levels approached baseline values over time (Supplemental Figure 5A). The expression of mitochondrial proteins encoded by mtDNA (COX-1) and nuclear DNA (SDHA), a measure of tissue mitochondrial content, were also significantly decreased in the kidneys of CLP mice treated with vehicle at 18 hours compared with mice subjected to sham or CLP surgery and treated with BAM15 (Supplemental Figure 5B).

PGC1α is activated by AMPK and sirtuin 1 (SIRT1) (26, 27). We found that CLP depressed PGC1α without significant changes in AMPK and SIRT1 levels; pretreatment with BAM15 increased expression of phosphorylated AMPK at 2 to 18 hours and SIRT1 at 18 hours (Supplemental Figure 5, C and D). Thus, BAM15 appears to activate AMPK during the early phase of sepsis-AKI and activates SIRT1 during the late phase of sepsis-AKI; this is followed by increasing mitochondrial biogenesis associated with PGC1α activation.

We also measured NAD^+^ in kidney at 18 hours after CLP. The activation of PGC1α by SIRT1 requires NAD^+^ (28). Levels of NAD^+^ as well as NADH were decreased in mice subjected to CLP and administered with vehicle, which did not change the NAD^+^/NADH ratio (Supplemental Figure 5, F–H). Early BAM15 pretreatment partially restored NAD^+^ levels (Supplemental Figure 5F), but did not alter NADH levels (Supplemental Figure 5G). Our data support an activation of Sirt1 by BAM15 through increased NAD^+^ levels, which would be expected to restore PGC1α levels.

PGC-1α promotes expression of many nuclear genes whose products are imported into mitochondria, including the mitochondrial transcription factor A (TFAM). TFAM is a mtDNA-binding protein that is essential for genome maintenance (29) and plays a central role in the mtDNA stress-mediated inflammatory response, including in AKI (30). BAM15 treatment increased TFAM from 6 hours after CLP (Supplemental Figure 5E) compared with CLP treated with vehicle. These results are consistent with BAM15 treatment enhancing expression of these mitochondrial biogenesis-related proteins.

### BAM15 reduced serum cytokines and immunosuppression.

**Figure 3:**
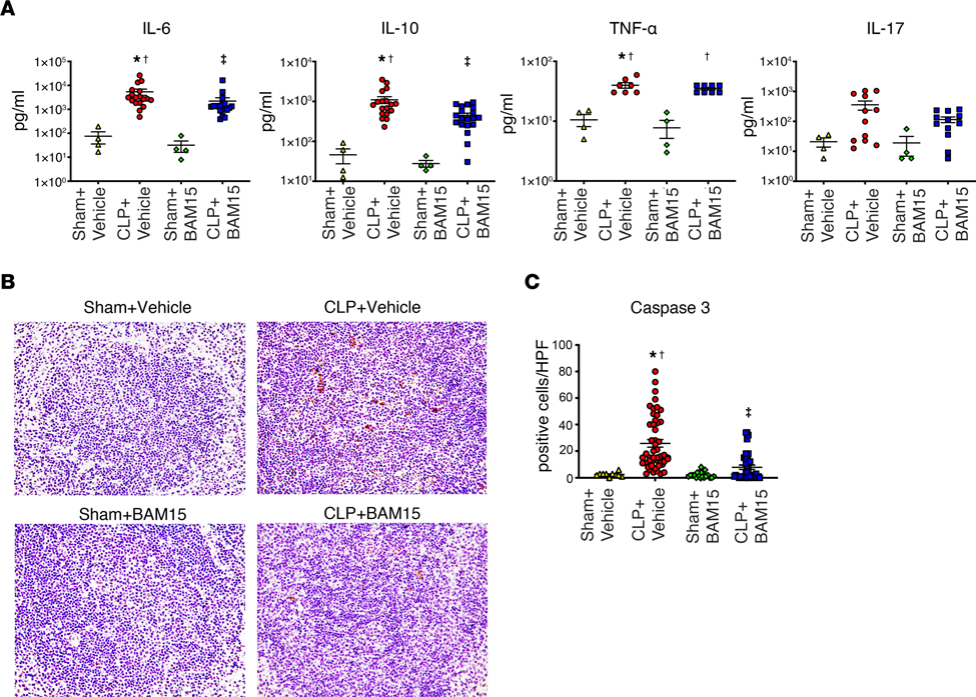
BAM15 inhibits production of some cytokines and splenic apoptosis. (**A**) Cytokines in serum at 18 hours after mice subjected to sham or CLP surgery were treated with vehicle (at 0 hours) or BAM15 (5 mg/kg, at 0 hours). *n* = 4 (sham+vehicle), *n* = 7–18 (CLP+vehicle), *n* = 4 (sham+BAM15), *n* = 8–19 (CLP+BAM15) mice**.** (**B**) Cleaved caspase-3 staining in the spleen at 18 hours after mice subjected to sham or CLP surgery were treated with vehicle (at 0 hours) or BAM15 (5 mg/kg, at 0 hours). Original magnification, ×400. (**C**) Positive cells of cleaved caspase-3 in the spleen of mice subjected to sham (*n* = 4 mice each, total 20 fields of 400×) or CLP surgery (8 mice each, total 40~50 fields of ×400) treated with vehicle (at 0 hours) or BAM15 (5 mg/kg, at 0 hours) at 18 hours. Data are represented as mean ± SEM. Dunn’s multiple-comparison test following Kruskal Wallis test. *Versus sham+vehicle, *P* < 0.05; ^†^versus sham+ AM15, *P* < 0.05; ^‡^versus CLP+vehicle, *P* < 0.05.

Sepsis causes both stimulation and suppression of the immune system. As expected, CLP increased serum IL-6, IL-10, TNF-α, and IL-17 (Figure 3A). BAM15 inhibited increases in both serum IL-6 and IL-10. Splenic apoptosis provides a measure of immunosuppression in mouse models of sepsis. CLP increased cleaved caspase-3–positive cells in spleen at 18 hours, which was decreased by pretreatment with BAM15 (5 mg/kg, i.p. at 0 hours after CLP) (positive cells: CLP+vehicle, 26 ± 2.9/high-power field [HPF] versus CLP+BAM15, 8.0 ± 1.6/HPF, *P* < 0.05) (Figure 3, B and C). Thus, BAM15 inhibited both the overproduction of systemic cytokines and immunosuppression after sepsis.

### BAM15 is neither bacteriostatic nor bactericidal.

Bacteria also have a membrane ETC (31); thus, BAM15 might directly affect bacterial energetics. We evaluated the effect of BAM15 on pathogens in the peritoneal cavity of septic mice. BAM15 (5 mg/kg) given to septic mice at 0 hours after surgery did not alter the number of bacterial colonies in blood or abdominal fluid assessed 18 hours after CLP. Blood bacterial counts were as follows: CLP+vehicle, 1.1 × 10^4^ ± 0.90 × 10^4^ CFU/ml versus CLP+BAM15, 0.10 × 10^4^ ± 0.053 × 10^4^ CFU/ml, *P* = 0.4. Abdominal bacterial counts were as follows: CLP+vehicle, 1.2 × 10^5^ ± 0.92 × 10^5^ CFU/ml versus CLP+BAM15, 6.9 × 10^5^ ± 6.6 × 10^5^ CFU/ml, *P* > 0.99 (Supplemental Figure 6A). We also tested bactericidal ability of BAM15 in vitro by directly adding BAM15 (0, 1, 5, 10, 20, and 50 μM) to a suspension of cecal material. BAM15 did not alter the number of bacterial colonies at any concentration (Supplemental Figure 6B). We conclude that BAM15 does not have bactericidal activity in vivo or in vitro.

### BAM15 inhibited cfmtDNA in mice.

**Figure 4:**
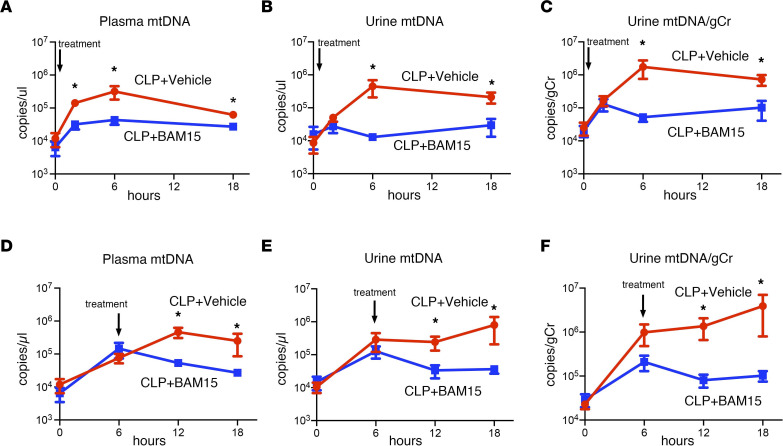
Both early and delayed BAM15 treatment decrease circulating mtDNA. (**A**–**C**) Time course of plasma mtDNA levels (**A**) and urine mtDNA (**B**) and urine mtDNA adjusted to creatinine excretion (**C**) at 18 hours after CLP (*n* = 8 each) mice were treated with vehicle (at 0 hours) or BAM15 (5 mg/kg, at 0 hours). (**D**–**F**) Time course of plasma mtDNA level (**D**) and urine mtDNA (**E**) and urine mtDNA adjusted to the creatinine excretion (**F**) in CLP mice (*n* = 8 each) treated with vehicle (at 6 hours) or BAM15 (5 mg/kg, at 6 hours).Data are represented as mean ± SEM. Analysis between groups at each time point was performed with Šidák’s multiple-comparison test following mixed-effects analysis. *P < 0.05.

While cfmtDNA is a biomarker of tissue injury in critically ill patients (32), it is unknown whether cfmtDNA levels can be used to predict and/or monitor drug efficacy. We evaluated the drug-biomarker relationship between BAM15 and cfmtDNA in male mice. We confirmed the correlation between past (8) and current mtDNA assays to quantitate mtDNA in plasma (Supplemental Figure 7, A–C). The new mtDNA assay did not detect bacterial DNA originating in blood of CLP mice (Supplemental Figure 7D). Plasma cfmtDNA increased from 2 to 18 hours after CLP; BAM15, given at the time of CLP surgery, reduced cfmtDNA 2 to 18 hours after CLP (plasma cfmtDNA at 2 hours: CLP+vehicle, 1.4 × 10^5^ ± 0.26 × 10^5^ copies/μl versus CLP+BAM15, 0.32 × 10^5^ ± 0.09 × 10^5^ copies/μl, *P* < 0.05) (Figure 4A). Urinary cfmtDNA also increased beginning 6 hours after CLP, and BAM15, given at the time of CLP surgery, reduced urinary cfmtDNA after CLP (mtDNA/g Cr in urine at 6 hours: CLP+vehicle, 1.8×10^6^ ± 1.0×10^6^ copies/μl versus CLP+BAM15, 0.52 × 10^5^ ± 0.14 × 10^5^ copies/μl, *P* < 0.05) (Figure 4, B and C).

The effect of BAM15 treatment on cfmtDNA was also observed in female mice (Supplemental Figure 1, D–F). Delayed treatment with BAM15 (6-hour delay) also inhibited both plasma and urine cfmtDNA (plasma cfmtDNA at 12 hours: CLP+vehicle, 4.6 × 10^5^ ± 1.6 × 10^5^ copies/μl versus CLP+BAM15, 0.53×10^5^ ± 0.12 × 10^5^ copies/μl, *P* < 0.05; urinary mtDNA/g Cr at 12 hours: CLP+vehicle, 1.4 × 10^6^ ± 0.70 × 10^6^ copies/g Cr versus CLP+BAM15, 0.81 × 10^5^ ± 0.26 × 10^5^ copies/g Cr, *P* < 0.05) (Figure 4, D–F). These results suggest that plasma and urinary cfmtDNA levels are responsive to BAM15 treatment in sepsis-AKI; thus, BAM15 and cfmtDNA are a potential drug-companion biomarker pair.

### BAM15 inhibits overproduction of mitochondrial superoxide and mtDNA release in cultured renal tubule cells exposed to serum from septic mice.

We established an in vitro model of septic kidney tubule cells by incubating mPPTCs with serum from septic mice obtained 18 hours after CLP. Overproduction of mtROS has been proposed as a pathophysiologic mechanism for sepsis-AKI (33). Mitochondrial uncouplers paradoxically decrease mitochondrial superoxide production by increasing the electron transfer rate, which decreases the dwell time for single electrons traversing the ETC (15–17).

**Figure 5:**
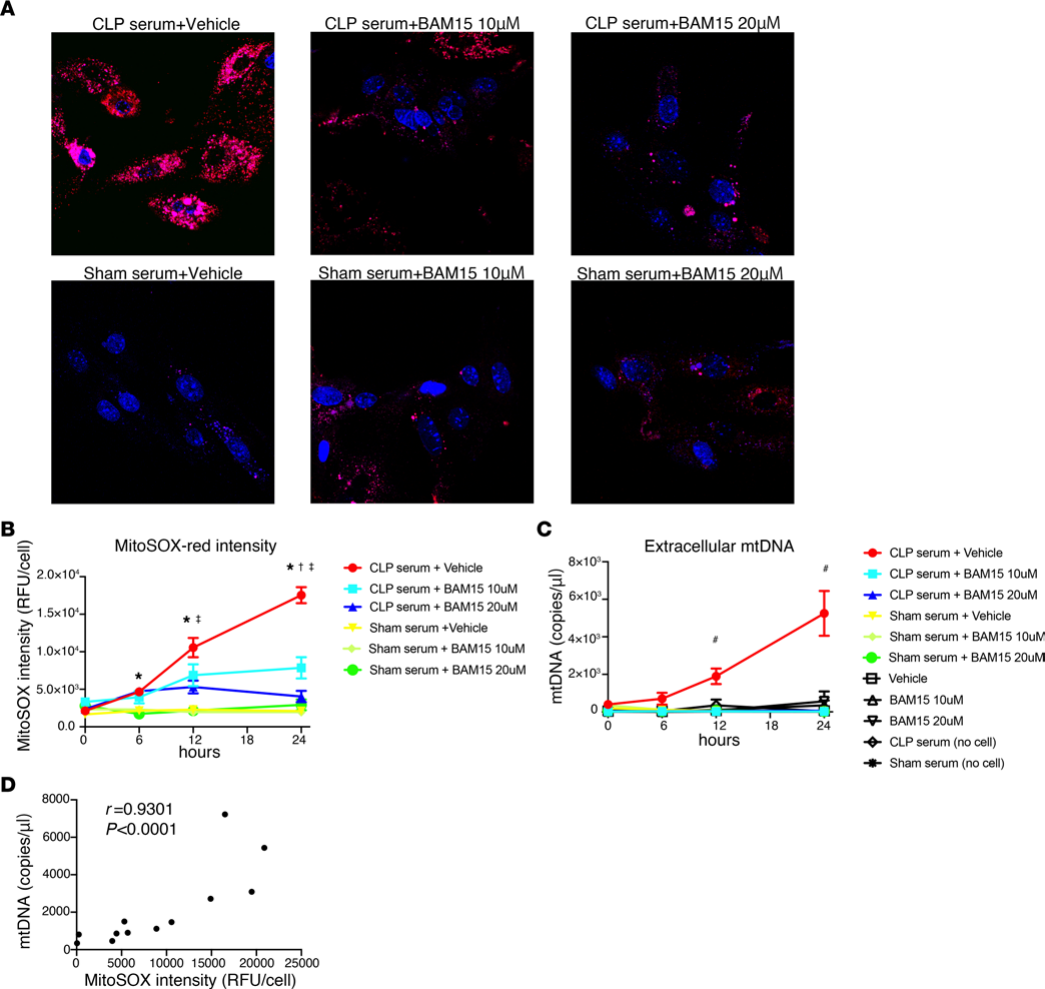
BAM15 inhibits production of mtROS in mPPTCs linked with decreasing mtDNA released from mPPTCs. (**A** and **B**) Serial live-cell imaging of mtROS in mPPTCs incubated with sham or CLP serum treated with vehicle or BAM15 (10 μM or 20 μM). (**A**) Representative images of mtROS of mPPTCs in each group at 24 hours after incubation. Red, MitoSOX Red; blue, Hoechst 33342. Original magnification, ×400. (**B**) Time course of fluorescence intensity of MitoSOX Red serially measured. Data are represented as mean ± SEM. Tukey’s multiple-comparison test following 2-way ANOVA test. *n* = 18–31 (CLP serum+vehicle), *n* = 16–24 (CLP serum+BAM15, 10 μM), *n* = 19–24 (CLP serum+BAM15, 20 μM), *n* = 18–32 (sham serum+vehicle), *n* = 9–13 (sham serum+BAM15, 10 μM), and *n* = 8–13 (sham serum+BAM15, 20 μM) for 3 biological replicates per condition. *Versus each serum from mice subjected to sham surgery group, *P* < 0.05; ^†^versus CLP serum+10 μM BAM15, *P* < 0.05; ^‡^versus CLP serum+20 μM BAM15, *P* < 0.05. (**C**) Time course of extracellular mtDNA levels of the supernatant shown in **B**. Controls were medium with CLP/sham serum or supernatants of PTCs treated with only vehicle/BAM15. ^#^CLP serum+vehicle versus each other group, *P* < 0.05. Tukey’s multiple-comparison test following 2-way ANOVA test. (**D**) Correlation between extracellular mtDNA level and the corresponding MitoSOX Red intensity in the mPPTCs incubated with CLP serum treated with vehicle. *n* = 8 from 0, 6, 12, and 24 hours on 2 biological replicates. *r* value is Pearson’s correlation coefficient.

To evaluate the effect of BAM15 on renal mtROS, we measured the production of mitochondrial superoxide using MitoSOX Red in mPPTCs coincubated with serum from CLP mice (“septic tubule cells”); measurements were made at 0, 6, 12, and 24 hours following the addition of mouse serum. Coincubation with septic serum increased MitoSOX Red intensity over time, compared with coincubation with control serum. BAM15 (either 10 μM or 20 μM) inhibited the overproduction of superoxide induced by CLP serum (Figure 5, A and B), with a significantly larger effect at 20 μM BAM15. These results suggest that BAM15 can act in part by decreasing mtROS production in tubule cells during sepsis. To evaluate whether mtDNA is released from septic tubule cells, we measured the appearance of cfmtDNA in the media of cultured tubule cells at 0, 6, 12, and 24 hours after incubation with septic serum. The concentration of cfmtDNA increased over time. Both 10 and 20 μM BAM15 inhibited cfmtDNA release from septic tubule cells (Figure 5C). Furthermore, cfmtDNA release correlated with MitoSOX Red generation in septic tubule cells (*r* = 0.93, *P* < 0.0001) (Figure 5D).

### BAM15 protects against mtDNA-induced kidney injury and systemic inflammation in vivo.

**Figure 6:**
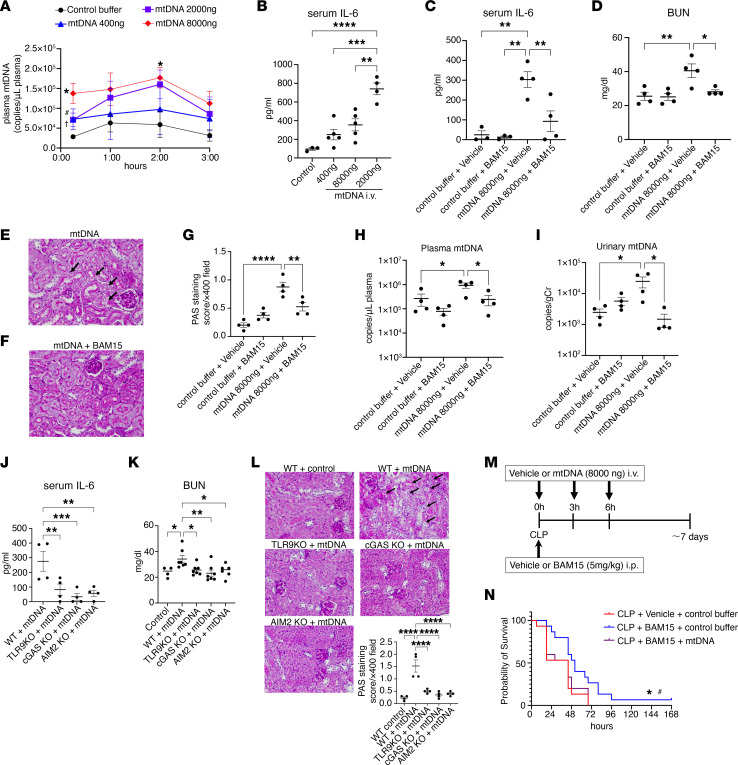
BAM15 effect on kidney injury and systemic inflammation caused by mtDNA as DAMPs in vivo. (**A**) Circulating mtDNA levels after injection of mtDNA (400 ng, 2,000 ng, or 8,000 ng) into naive mice. *n* = 3–4 each. Data are represented as mean ± SEM. *P < 0.05, mtDNA, 8,000 ng versus control; ^#^*P* < 0.05, mtDNA, 2,000 ng versus control; ^†^*P* < 0.05, mtDNA, 400 ng versus control, unpaired *t* test. (**B**) IL-6 levels in serum at 3 hours after injection of mtDNA (400, 2,000, or 8,000 ng) into naive mice. *n* = 3–5 each. Data are represented as mean ± SEM. *P < 0.05; **P < 0.01; ***P < 0.001; ****P < 0.0001, Tukey’s multiple-comparison test following 1-way ANOVA. (**C**–**I**) BAM15 effect (5 mg/kg, i.p., at 0 hours) at 3 hours after injection of mtDNA (8,000 ng) into naive mice on serum IL-6 level (**C**), BUN (**D**), PAS staining (**E** and **F**), and scoring (**G**) of kidney cortex, and mtDNA level in plasma (**H**) and urine (**I**). Original magnification, ×400. *n* = 3–5 each. Data are represented as mean ± SEM. *P < 0.05; **P < 0.01; ****P < 0.0001, Šidák’s multiple-comparison test following 1-way ANOVA. (**J**–**L**) Serum IL-6 levels (**J**), BUN (**K**), and PAS staining and scoring (**L**) in WT, TLR9-KO, cGAS-KO, and AIM2-KO mice at 3 hours after injection of mtDNA (8,000 ng). *n* = 4 each for IL-6 and PAS staining. *n* = 6 for BUN. Data are represented as mean ± SEM. *P < 0.05; **P < 0.01; ***P < 0.001. Šidák’s multiple-comparison test following 1-way ANOVA. (**M**) Study design for survival study of mtDNA (8,000 ng) injection at 0, 3, and 6 hours after CLP surgery. (**N**) Kaplan-Meier curves of CLP mice treated for 7 days with vehicle or BAM15 (5 mg/kg, at 0 hours) following injection of mtDNA (8,000 ng) or control buffer. *n* = 15 each. log-rank test. *P < 0.05, CLP+vehicle+control versus CLP+BAM15+control; ^#^*P* < 0.05, CLP+BAM15+mtDNA versus CLP+BAM15+control buffer.

To investigate the functional role of mtDNA in vivo, we first injected mtDNA (400 ng, 2000 ng, or 8000 ng) into normal CD-1 mice and then measured circulating mtDNA and other relevant biomarkers. Plasma mtDNA increased 15 minutes after injection at all doses, peaking at 2 hours after injection (Figure 6A). The inflammatory marker serum IL-6 levels were increased at 3 hours in a dose-dependent manner (Figure 6B). We detected kidney dysfunction and vacuolization in tubular cells 3 hours after injection of 8,000 ng mtDNA (Figure 6, D–G). BAM15 overcame the IL-6 effect and kidney toxicity of exogenous mtDNA in nonseptic mice (Figure 6, C–G). Plasma and urine mtDNA levels were reduced by BAM15 at 3 hours after mtDNA injection, suggesting that BAM15 inhibits further release of mtDNA into the systemic circulation (Figure 6, H and I). The changes following mtDNA injection at 3 hours were inhibited in TLR9-KO, cGAS-KO, and AIM2-KO mice (Figure 6, J–L), suggesting that mtDNA-induced systemic and kidney injury occurred via all 3 major DNA-sensing pathways: TLR9, cGAS, and AIM2. We then addressed whether injection of mtDNA could overcome the BAM15 effect in CLP mice. Because of the short circulating half-life of mtDNA, we injected 8,000 ng of mtDNA (or vehicle) 3 times (at 0, 3, and 6 hours after CLP) into CLP mice treated with BAM15 or vehicle. The survival rate of CLP in all groups was low due to frequent anesthesia and injections; however, the highest dose of injected mtDNA overcame the beneficial effect of BAM15 on survival of CLP mice (Figure 6, M and N).

### BAM15 inhibits mtDNA-induced overproduction of mtROS and mtDNA release in vitro.

Purified mtDNA from the liver of mice subjected to CLP increased tubule cell mtROS and increased release of mtDNA, whereas BAM15 inhibited both mtROS generation and mtDNA release (Supplemental Figure 8, A–C). mtDNA-induced mtROS generation was inhibited in mPPTCs purified from TLR9-KO, cGAS-KO, and AIM2-KO mice, which are known receptors for mtDNA (7) (Supplemental Figure 8, D–H). These results indicate that BAM15 inhibits mtROS, which is generated by mtDNA via the TLR9, cGAS, and AIM2 pathways. On the other hand, exogenous superoxide (1, 10, and 100 μM KO_2_) promoted mtDNA release from tubule cells, which was inhibited by BAM15 (Supplemental Figure 8, I and J). These results suggest BAM15 may act to suppress a vicious cycle between mtROS and mtDNA.

### BAM15 requires neutrophils to improve survival and reduce kidney injury from sepsis.

**Figure 7:**
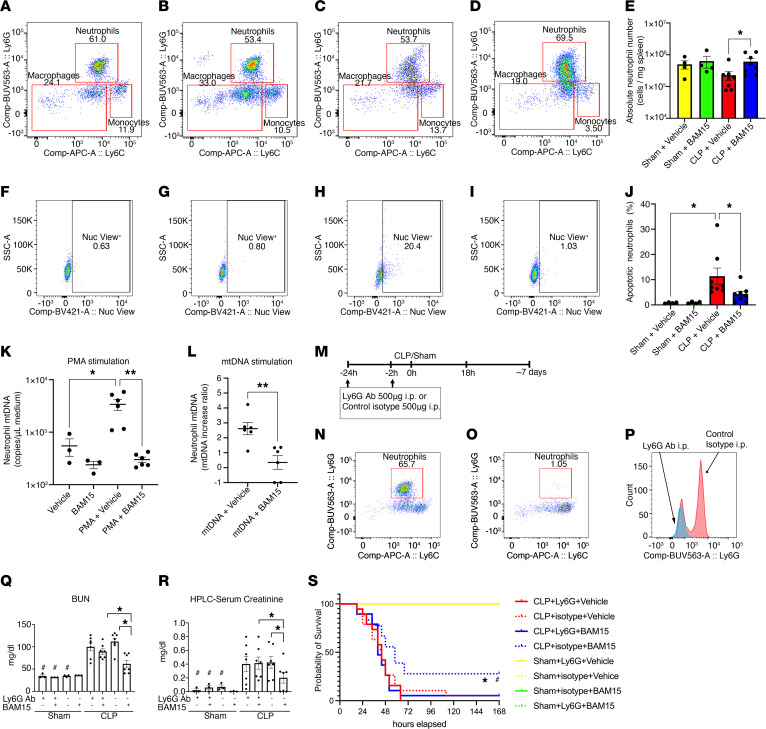
BAM15 decreases neutrophil apoptosis and mtDNA release; neutrophil depletion counteracted BAM15 benefits. (**A**–**D**) Gating of Ly6G^+^ neutrophils in granulocytes (CD45^+^CD11b^+^) of spleen at 18 hours of sham+vehicle (**A**), sham+BAM15 (**B**), CLP+vehicle (**C**), and CLP+BAM15 (5 mg/kg, at 0 hours) (**D**). (**E**) Absolute neutrophil number in each group. Sham groups, *n* = 4 each; CLP groups, *n* = 8 each. Each circle represents a log-transformed average of duplicated samples. (**F**–**I**) Apoptotic cells in neutrophils of spleen at 18 hours of sham+vehicle (**F**), sham+BAM15 (**G**), CLP+vehicle (**H**), and CLP+BAM15 (**I**), and percentage of these apoptotic neutrophils in each group (**J**). *n* = 4 for each sham group; *n* = 8 for each CLP group. *P < 0.05. (**K** and **L**) Extracellular mtDNA in medium of Ly6G^+^ neutrophils treated with PMA (**K**) or mtDNA (25 μg/ml) (**L**) with or without BAM15. Data are represented as mean ± SEM. Holm-Šidák multiple-comparison test following 1-way ANOVA (**E**, **J**, **K**); *t* test (**L**). *P < 0.05; **P < 0.001. (**M**) Study design of CLP with neutrophil depletion. (**N**–**P**) Ly6G^+^ neutrophil population in isotype control–treated (**N**) or Ly6G Ab–treated (**O**) spleen at 18 hours after sham surgery, and histogram of Ly6G expression (**P**). BUN (**Q**); Serum creatinine (**R**). Sham groups, *n* = 3 each; CLP groups, *n* = 7 each. Data are represented as mean ± SEM. Šidák’s multiple-comparison test following 1-way ANOVA. ^#^*P* < 0.05, CLP versus sham in each treatment group; *P < 0.05, between CLP groups. (**S**) Kaplan-Meier curves for 7 days of mice subjected to sham or CLP surgery treated with vehicle or BAM15 (5 mg/kg at 0 hours) following Ly6G Ab or isotype control treatment. Sham groups, *n* = 3 each; CLP groups, *n* = 20 each. log-rank test. *P < 0.05, CLP+isotype+vehicle versus CLP+isotype+BAM15. ^#^*P* < 0.05, CLP+Ly6GAb+BAM15 versus CLP+isotype+BAM15.

Because we demonstrated that kidney tubule cells can be a source of mtDNA, we searched for additional/alternative sources of circulating mtDNA as well as potential inflammatory/immune cell targets for BAM15. By histological staining, we determined that CLP-induced splenic apoptosis was decreased by BAM15, but we did not know what cell type was responsible for this result. We measured the absolute number of prevalent immune cell types by multicolor flow cytometry (B [CD3^–^CD19^+^], T [CD3^+^CD19^–^], NK-T [CD3^–^CD19^–^NK1.1^+^], neutrophils [CD11b^+^Ly6G^+^], monocytes [CD11b^+^Ly6C^+^], macrophages [CD11b^+^Ly6G^–^Ly6G^–^], and plasmacytoid DCs [pDC] [CD45R/B220^+^CD11c^+^]) in the spleen of mice subjected to sham (*n* = 4/each) or CLP surgery (*n* = 8/each) at 18 treated with vehicle or BAM15 (5 mg/kg, 0 hours). We found that numbers of splenic neutrophils were significantly decreased in CLP (vehicle treated) compared with CLP treated with BAM15 (Figure 7, A–E), with reciprocal changes in the percentage of apoptotic neutrophils (Figure 7, F–J). We did not see dramatic changes in other cell types. To investigate the effect of circulating mtDNA and BAM15 on neutrophils, Ly6G^+^ neutrophils were isolated from mouse spleen and stimulated by PMA or mtDNA with or without cotreatment of BAM15 for 3 hours. Supernatant mtDNA increased in both PMA- and mtDNA-stimulated neutrophils, and both were inhibited by BAM15 (Figure 7, K and L). Interestingly, mtDNA release stimulated by both PMA and mtDNA was inhibited in TLR9-KO, cGAS-KO, and AIM2-KO neutrophils (Supplemental Figure 9, A and B).

Next, to determine whether BAM15 requires neutrophils for its beneficial effects, we depleted neutrophils by injection of Ly6G-specific mAb (Figure 7, M–P). BAM15 (given at 0 hours) improved survival in isotype-injected mice but not in neutrophil-depleted CLP mice (Figure 7S). Similarly, BAM15 improved kidney function (at 18 hours) of isotype-injected mice but not in neutrophil-depleted CLP mice (CLP+Ly6Gab+BAM15 versus CLP+control+BAM15: serum creatinine, 0.41 ± 0.09 versus 0.20 ± 0.08 mg/dl, *P* < 0.05; BUN: 89.3 ± 6.10 versus 61.7 ± 8.02 mg/dl, *P* < 0.05) (Figure 7, Q and R). These results support the hypothesis that the protective effects of BAM15 on survival and kidney injury are mediated by neutrophils.

### BAM15 inhibited neutrophil infiltration into kidney.

**Figure 8:**
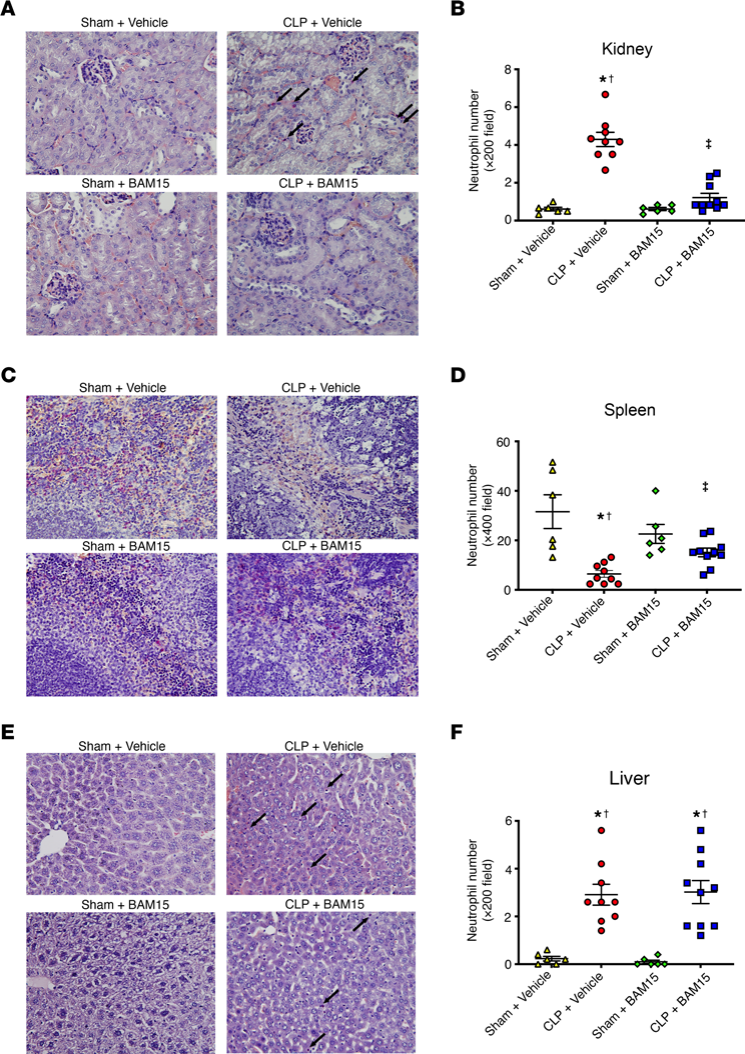
Neutrophil infiltration in kidney, spleen, and liver in septic mice. (**A**–**F**) Representative images and number of neutrophils (arrows) in kidney (**A**), spleen (**C**), and liver (**E**) 18 hours after sham/CLP mice were treated with vehicle or AM15 (5 mg/kg, at 0 hours) using naphthol AS-D chloroacetate esterase staining. Neutrophils are stained pink. Original magnification, ×400. Data are represented as mean ± SEM. *n* = 6 mice for sham surgery groups; *n* = 9–10 mice for CLP groups. Neutrophils were counted in ×200 fields (*n* = 5) for kidney (**B**) and liver (**F**), and ×400 fields (*n* = 5) for spleen (**D**) and averaged per mouse. Dunn’s multiple-comparison test following Kruskal-Wallis test. *Versus sham+vehicle, *P* < 0.05; ^†^versus sham+BAM15, *P* < 0.05; ^‡^versus CLP+vehicle, *P* < 0.05.

We confirmed the decrease in splenic neutrophils at 18 hours after CLP by histochemical staining of neutrophils, whereas BAM15 (5 mg/kg i.p. at 0 hours after CLP) inhibited the decline of neutrophil staining (Figure 8, A and B). In contrast, neutrophil numbers increased in the kidney at 18 hours after CLP (sham+vehicle versus CLP+vehicle: 0.61 ± 0.09 versus 4.3 ± 0.38 in ×200 field, *P* < 0.05), but not in kidneys of CLP treated with BAM15 (sham+BAM15 versus CLP+BAM15: 0.61 ± 0.08 versus 1.2 ± 0.23 in ×200 field, *P* < 0.05) (Figure 8, C and D). Moreover, BAM15 treatment did not inhibit neutrophil infiltration in the liver at 18 hours after CLP (Figure 8, E and F).

## Discussion

The pathogenesis of multiorgan failure in sepsis is complex. Multiple pathogen–associated molecular patterns (PAMPs) and DAMPs released from injured tissues in animals and humans (34) cause a runaway inflammatory response, which propagates additional organ damage and can be immunosuppressive (35). Because of the multiplicity of potentially parallel targets, a targeted therapeutic approach for an individual DAMP/PAMP might be ineffective; therapy may instead need to target a common downstream node. We and others previously showed mice lacking TLR9 or treated with a TLR9 inhibitor were protected from sepsis mortality and AKI (8, 36, 37), and a TLR9 ligand mtDNA was released during early sepsis and contributes to cytokine production and AKI (8). It is not known how well the mtDNA pathway is amenable to drug targeting, as cGAS and AIM2 can also sense mtDNA (38). In this study, we tested the effects of BAM15, a mitochondrial protectant. We found that (a) BAM15 improved survival and AKI after sepsis, even with a 12-hour treatment delay; (b) levels of 1 DAMP, mtDNA, were diminished by BAM15 treatment in vivo and in vitro; (c) mtDNA caused tissue and cell injury that were reversed by BAM15; (d) BAM15 reduced kidney injury by reducing neutrophil infiltration and tubule ROS/RNS generation and improving mitochondrial biogenesis; (e) BAM15 actions were abrogated in neutrophil-depleted mice; and (f) BAM15 inhibited a positive feedback loop involving mtDNA and mtROS. We conclude that BAM15 and mtDNA are a drug-companion biomarker combination for treatment of sepsis. mtDNA is an early efficacy biomarker that can also mediate tissue injury.

### BAM15 improved survival and AKI after sepsis, even with a 12-hour treatment delay.

Effective therapeutics are needed for sepsis and septic shock. Efficacy with delayed treatment is critical, as the early stages of human sepsis may begin out of hospital and are often difficult to diagnose. In this study, the mitochondrial uncoupler BAM15 improved survival when given at the time of CLP surgery and also when treatment was delayed 6 and 12 hours (Figure 1 and Supplemental Figure 2), well after the animals exhibited clinical signs of sepsis — generally around 5 to 6 hours (39). In previous studies of other agents (36, 40, 41), delayed administration (“treatment”) is often less effective than when given at the time of CLP surgery (“prevention”). BAM15 joins a short list of potential treatments for sepsis that improve survival, rather than just delaying death, in animal models of sepsis (42–45).

BAM15 had other beneficial systemic effects during sepsis. BAM15 improved systemic hemodynamics (reducing hypotension, increasing HR and BT), decreased several pro- and antiinflammatory systemic cytokines (IL-6, IL-10), and reduced splenic apoptosis (a marker of later immunosuppression; ref. 46). BAM15 might contribute to inhibiting persistent immunoparalysis in sepsis.

BAM15 improved survival and AKI, but did not protect against acute liver injury (Figure 1). Interestingly, BAM15 inhibited neutrophil infiltration after sepsis into the kidney, but not the liver (Figure 8, E and F), and it increased splenic neutrophils (Figure 8, C and D). Further studies are needed for unraveling the tissue-specific effects of BAM15 on neutrophil infiltration (47) and survival.

### Circulating and urinary levels of mtDNA were diminished by BAM15 treatment in vivo and in vitro.

We and others have shown that circulating mtDNA is increased in ischemia/reperfusion, sepsis, and COVID-19 (10–12, 48, 49). mtDNA predicts mortality (48) and is a marker of tissue injury (49). For example, mtDNA increased 10- to 100-fold in plasma of COVID-19 patients and correlated with severity of disease. Purified circulating DNA (including mtDNA and nuclear DNA) increased mtROS in mouse kidney tubules in vitro, with purified DNA accounting for the entire effect of plasma (11). In this study, we found that circulating mtDNA and urinary mtDNA are increased very early in sepsis in both male and female mice (Figure 4 and Supplemental Figure 1). mtDNA increase (2 to 6 hours) precedes detection of increased creatinine (typically 12 to 18 hours) (23, 41). Urinary mtDNA (6 to 24 hours) was also more sensitive than BUN (24 hours) in detecting subclinical renal injury in an ischemia/reperfusion model, although a detailed time course was not measured (49). Small clinical studies show that high levels of circulating mtDNA, especially urinary mtDNA, predict AKI and mortality in critically ill patients (50) and are associated with more severe sepsis-AKI (51). More importantly, we also found that BAM15 rapidly decreased circulating and urinary mtDNA in vivo in both male and female CLP mice early in sepsis (Figure 4 and Supplemental Figure 1). We confirmed these effects using in vitro sepsis models. BAM15 also decreased mtDNA release that had been stimulated in vitro by septic serum in mouse kidney tubules and by PMA in mouse neutrophils (Figure 5 and Figure 7K). The rapid response of mtDNA to BAM15 treatment suggests that mtDNA could be used as a drug-efficacy biomarker (see below). The level of mtROS or mtDNA might be useful in selecting septic patients likely to benefit from BAM15. The monitoring of mtROS or mtDNA followed by BAM15 treatment might be able to predict “target engagement,” and hence predict efficacy and outcome. Future development of a rapid assay of mtROS and/or mtDNA would aid in using BAM15-mtDNA as a drug-companion biomarker pair for precision medicine therapy in sepsis. Does this close correspondence between drug effect and biomarker have a mechanistic basis?

### mtDNA causes tissue and cell injury that are reversed by BAM15.

Septic serum contains many DAMPs that are merely markers of tissue injury, but which are also mediators of disease? We previously showed that infusion of damaged mitochondria produced a sepsis-like histologic kidney damage that was reversed by DNase, suggesting that mtDNA is toxic (8). In a study of patients with COVID-19, we found that human mtDNA caused proximal tubule damage in vitro (11). However, the ability of mtDNA to cause direct animal/tissue toxicity has not been well studied. We found that injection of mtDNA increased an inflammatory cytokine (IL-6) and caused kidney injury, which was inhibited by deletion of any one of the three classic mtDNA receptors (TLR9, cGAS, and AIM2) (Figure 6, C–G and J–L). The mtDNA-stimulated IL-6 increase and kidney injury were both reduced by BAM15 (Figure 6, C–G). mtDNA also stimulated cultured proximal tubule cells, resulting in mtDNA release, which was also reduced by BAM15 (Supplemental Figure 8). mtDNA increased mtROS generation via TLR9, cGAS, and AIM2 pathways and, interestingly, promoted further mtDNA release (Supplemental Figure 8). Both mtROS and mtDNA effects were inhibited by BAM15. mtDNA is reported to promote mtDNA release and neutrophil extracellular trap (NET) formation via a TLR9 pathway in lung transplantation (52). We also found that mtDNA promoted release of neutrophil mtDNA, which was inhibited by BAM15 (Figure 7L). The BAM15 treatment effect on CLP survival could be overcome by frequent injections of large amounts of exogenous mtDNA (Figure 6N). Thus, BAM15 has a therapeutic window that can be overcome by large amounts of toxic mtDNA. The exact therapeutic window is hard to calculate because our model includes both endogenous and exogenous mtDNA, and the animals were also adversely affected by frequent anesthesia and injections. Collectively, our results indicate that mtDNA is toxic in vivo and in vitro to proximal tubule cells and neutrophils; thus, mtDNA is also a mediator of disease.

### BAM15 reduces kidney injury by reducing neutrophil infiltration, reducing tubule ROS generation, and improving mitochondrial biogenesis.

The role of mitochondrial damage in AKI, sepsis, and sepsis-AKI is well studied (20, 26, 51, 53, 54); reduced mitochondrial mass has been reported in the kidneys of patients with sepsis-AKI (55). We found that BAM15 protected against sepsis-AKI, assessed by measures of glomerular filtration (serum BUN and creatinine), histologic damage indicative of sepsis (tubular vacuolization, tubule dilation), and tubule hypoxia typical of sepsis (pimonidazole staining; Supplemental Figure 3, B and C). The effects on renal function were also replicated in female mice (Supplemental Figure 1B). BAM15 has been shown to protect the kidney from cold storage–induced damage (13) and in a kidney ischemia/reperfusion model (14). In the latter model, BAM15 prevented tubular necrosis and vascular pooling (14). The actual mechanism of BAM15’s effect on the kidney had not been determined. We found several protective pathways. First, BAM15 inhibited sepsis-induced neutrophil infiltration into the kidney (Figure 8, A and B). Neutrophil infiltration into ischemic and septic mouse kidney in sepsis is well described (56–59). Neutrophils migrate into the renal interstitium and are thought to promote kidney injury through the secretion of cytokines, ROS, and proteases (60). Whereas blocking neutrophil infiltration protected mice from ischemia/reperfusion and cisplatin forms of AKI (61, 62), neutrophil depletion does not prevent kidney injury in CLP sepsis-induced kidney injury (63). We also found that neutrophil depletion itself did not affect kidney function in the group not treated with BAM15 (Figure 7, Q and R). BAM15 inhibited neutrophil infiltration into the septic kidney in mice when neutrophils were intact; however, BAM15 did not improve kidney function if systemic neutrophils were depleted. As is discussed below, neutrophils have both protective and tissue-destructive functions. BAM15 may tip the balance when neutrophils are present, for example, by reducing release of toxic mtDNA.

Second, BAM15 had a direct effect on tubule cells. BAM15 decreased RNS in septic kidneys (Figure 2, C–F), decreased mitochondrial superoxide production in septic renal cells (Figure 5), and decreased mitochondrial superoxide production in mtDNA-treated renal cells (Supplemental Figure 8). Thus, BAM15 can act downstream of mtDNA. Third, BAM15 normalized pathways that would inhibit mitochondrial biogenesis (Supplemental Figure 5). Single electrons in the ETC exist only transiently at the redox centers, and the dwell time for single electrons in an unstable state increases the likelihood of superoxide production (17). Mitochondrial uncouplers decrease mitochondrial superoxide production by stimulating faster electron transfer through the electron transfer chain. Beyond inhibition of mtROS overproduction, BAM15 also altered several mediators of mitochondrial biogenesis, including PGC-1α (early and late after CLP), pAMPK (early), SIRT1 (late), and NAD^+^ (late) (Supplemental Figure 5). PGC-1α, activated by pAMPK and Sirt-1 (64), is a key transcriptional regulator of mitochondrial function, biogenesis, and respiration in many tissues, including kidney (65). BAM15 treatment is reported as an AMPK activator in other cell types (18, 66). We also showed that BAM15 increased TFAM expression, which is downstream of PGC1α, in kidney during an early phase of sepsis. TFAM depletion may promote leakage of mtDNA into the cytosol, followed by activation of innate immune response (67). Zhao et al. recently showed that mtROS promoted ischemia/reperfusion AKI by suppressing TFAM transcription and enhancing protease-mediated TFAM degradation (53). Thus, decreased renal mtROS by BAM15 may promote TFAM expression in the septic kidney and inhibit sepsis-AKI progression.

In summary, BAM15 could limit renal injury by (a) inhibiting neutrophil infiltration and/or inhibiting indirect actions by neutrophils (release of mtDNA, for example), (b) directly protecting proximal tubule cells from circulating DAMPs (mtDNA) by reducing production of ROS/reactive nitrogen species, and (c) improving mitochondrial biogenesis.

### BAM15 actions were abrogated in neutrophil-depleted mice.

In contrast with the kidney, where sepsis increased neutrophil infiltration (Figure 8, A and B), sepsis decreased neutrophils in the spleen (Figure 8, C and D) and increased apoptotic neutrophils. Sepsis shortens neutrophil life span in patients, and shorter neutrophil life span correlates with higher mortality (68). BAM15 inhibited neutrophil mtDNA release in vivo, reduced neutrophil apoptosis, and increased net neutrophil survival in septic spleen (Figure 7, A–L). Neutrophils are essential to BAM15’s effects on survival and kidney injury following CLP (Figure 7S). That neutrophils could mediate the systemic benefit of BAM15 on survival seems paradoxical, as neutrophil depletion does not alter overall survival after CLP (63, 69) or for renal injury after CLP (63) (Figure 7S). The most straightforward explanation is that BAM15 tips the balance so that neutrophils are more protective than detrimental, as neutrophils can have an extensive number of both beneficial and deleterious effects, including bacterial killing, release of cf DNA, NET formation (trapping bacteria and promoting vascular thrombosis), cytokine/chemokine release, and interacting with other immune cells. Based on the results of this study, bacterial killing is unaffected by BAM15, at least at 18 hours after CLP. Neutrophils are a major source of cf DNA in sepsis (70) and during COVID-19 (11). Neutrophil-derived cfDNA correlates with increased mortality and severity in COVID-19 patients (11). In our results, BAM15 inhibited release of mtDNA from neutrophils stimulated by either PMA or mtDNA. BAM15 most likely interrupts a destructive, pathogenic feed-forward loop in neutrophils, while simultaneously allowing neutrophils to protect against infection. Like many other agents either in use (glucocorticoids, glucose control, and fluids) or that have been tested for use in sepsis, neutrophils have both beneficial and harmful aspects, and effective treatments will need to mitigate harmful aspects without compromising beneficial aspects. In summary, neutrophils appear to be critically involved in the beneficial effects of BAM15.

### BAM15 inhibits a positive feedback loop involving mtDNA and mtROS.

We found in several in vitro and in vivo settings that septic serum increased mtDNA release. In cultured proximal tubule cells, serum from septic mice increased mtROS and also increased extracellular mtDNA concentration in culture medium, and both were reduced by BAM15 (Figure 5). Septic serum contains bacteria, PAMPs, DAMPS (including DNA and mtDNA), cytokines, chemokines, etc. To rule out a contribution of bacteria, we filtered the serum. To more closely examine mtDNA, we purified mtDNA from septic liver mitochondria (see Supplemental Methods). We also obtained similar results when cells were stimulated with either septic serum or purified mtDNA, which increased mtROS via TLR9, cGAS, and AIM2 pathways and released additional mtDNA. Finally, ROS alone can directly induce mtDNA release, as exogenous superoxide (KO_2_) also increased extracellular mtDNA, which was then reduced by BAM15 (Supplemental Figure 8). The tight correlation between mtDNA concentration and fluorescence intensity of MitoSOX Red supports a mechanistic relationship between ROS overproduction and mtDNA release. In this model, mtDNA increases ROS (via TLR9, cGAS, and AIM2 pathways), which causes cell stress/damage and mtDNA release. The additional mtDNA would cause additional ROS production, etc., thus forming a positive feedback loop involving mtROS and mtDNA that can be suppressed by BAM15. Note that the released mtDNA could be recognized by TLR9 either intracellularly or extracellularly (7, 8, 71). While other existing biomarkers of AKI (e.g., KIM-1, L-FABP) are associated with excessive ROS and hypoxia, respectively, and are acute markers of tissue injury (72, 73), they are not known to mediate further damage. Indeed, KIM-1–mediated phagocytosis of apoptotic cells protects the kidney after acute injury by downregulating innate immunity and inflammation (74).

Thus, BAM15 interrupts a positive feedback loop involving mtDNA that mediates tissue injury; therefore, BAM15 and circulating and/or urinary mtDNA form a drug companion biomarker pair that could be clinically useful. The rapid increase in mtDNA after the initiation of sepsis could serve as a detection marker of early sepsis as well as assisting with identification of patients who could benefit from BAM15 treatment. The rapid decrease of mtDNA upon treatment with BAM15 could be useful as a drug-efficacy biomarker.

### Limitations.

There are several limitations to the present study. We only studied a single dose (5 mg/kg i.p.) of BAM15, based on the published literature (14). To investigate optimal dosing, further pharmacokinetic studies are warranted. We only studied the mechanism of action of BAM15 in relatively early sepsis (mostly 0 hours pretreatment). Although BAM15 was still effective when treatment was delayed by 12 hours, we cannot eliminate the possibility that there is a shift in mechanism during delayed treatment. We did not perform a sex-stratified analysis because it is known that female septic mice have a better survival rate than male septic mice (75, 76) that is mainly explained by sex hormones (77). Also, it is impossible to blind the surgeon to the sex of the animal. Biological sex might affect the response to drugs in a CLP model (78). Finally, extensive further study, outside the scope of the current paper, would be needed to unravel how BAM15 alters neutrophil function(s). Also, we cannot eliminate the participation of other cell types in the action of BAM15.

### Conclusion.

In conclusion, BAM15 administration either before or 6 to 12 hours after induction of sepsis decreased mortality in a clinically relevant animal model of abdominal sepsis that manifests bacteremia, unstable hemodynamics, and high mortality despite treatment with volume resuscitation and broad-spectrum antibiotics. BAM15 improved kidney injury and attenuated splenic apoptosis, although it did not have effects on other organs or some cytokines. mtDNA was an early circulating and urinary marker of sepsis, reduced by BAM15, and toxic in vivo and in vitro. We established a tight coupling between the BAM15 effect on mtROS and mtDNA using in vitro assays and effects on mitochondrial homeostasis. The beneficial BAM15 effects on survival and kidney injury were reduced by neutrophil depletion. We conclude that BAM15 may be an effective preventive and therapeutic agent in sepsis, linking animal survival, mtDNA, kidney tubule damage, and neutrophils. mtDNA is both a marker and mediator of tissue injury. Thus, BAM15 and mtDNA are mechanistically linked, and they may form a drug-companion biomarker pair as part of an evolving precision-medicine approach to initiating and monitoring the treatment of patients with sepsis.

## Methods

### Animals.

All animals had free access to water and chow throughout the study. We followed the recent recommendations on minimum quality threshold in preclinical sepsis studies (MQTiPSS) (79).

### Statistics.

Data are presented as mean ± SEM. Normality and log normality were analyzed by the Shapiro-Wilks test. To determine statistically significant differences between independent continuous variables, a 2-tailed Student’s *t* test was applied when comparing 2 groups for which both data sets had a normal distribution. Comparison of 3 or more groups was assessed by 1-way ANOVA, and the influence of 2 different independent variables (i.e., time point and group) on an outcome was assessed by 2-way ANOVA when data were normally distributed. Mixed-effects analysis was performed instead of 2-way ANOVA to handle missing data. Dunn’s multiple-comparison test was used with the Kruskal-Wallis test when the data were not normally distributed. As a post hoc test, Tukey’s multiple-comparison test was used for both 1-way and 2-way ANOVA analyses. Šidák’s multiple-comparison test was used for pairwise comparisons of groups. Pearson’s correlation coefficient was used to analyze associations between 2 continuous variables. The prediction ability for mortality data was assessed using receiver operating characteristic (ROC) curve analysis and the log-rank test. A *P* value of less than 0.05 was considered significant. All statistical analyses were performed using Prism, version 8.4.3 (GraphPad Software).

### Study approval.

We followed NIH guidelines for the use and treatment of laboratory animals, and the NIDDK Animal Care and Use Committee approved all procedures.

Further details are provided in Supplemental Methods.

## Author contributions

NT, PSTY, and RAS conceived and designed research. NT, TT, TY, NH, and XH performed experiments. NT analyzed data. NT, TT, PSTY, and RAS interpreted the results of experiments. NT prepared the figures. NT drafted the manuscript. NT, TT, PSTY, and RAS edited and revised the manuscript. NT, TT, TY, NH, XH, PSTY, and RAS approved the final version of manuscript.

## Supplementary Material

Supplemental data

## Version 1 02/09/2023

In-Press Preview

## Version 2 04/03/2023

Electronic publication
