# Supplementary material for: BAM15 treats mouse sepsis and kidney injury, linking mortality, mitochondrial DNA, tubule damage, and neutrophils
Source: J Clin Invest. 2023 Apr 3;133(7):e152401. doi: 10.1172/JCI152401 (PMC10065071; doi:10.1172/JCI152401)
Supplement: Supplemental data [file jci-133-152401-s009.pdf]

Supplemental Methods

Animals

TLR9 knockout mice were obtained by crossing male and female of heterozygous for $Tlr9^{em1.1Ldm}$ (strain:034449, Jackson Laboratory, Bar Harbor, MA). Genotyping for TLR9 was processed by TransnetYX, Inc. (Cordova, TN). cGAS knockout and AIM2 knockout mice were obtained by breeding of B6(C)-$Cgas^{tm1d(EUCOMM)Hmgu}$/J (strain:026554), and B6.129P2-$Aim2^{Gt(CSG445)Byg}$/J (strain:013144) respectively (Jackson Laboratory). C57BL/6J (strain: 000664) as wild type for these 3 KO mice were also obtained from Jackson Laboratory.

Animal models

Male or female CD-1 mice (Charles River Laboratories, Wilmington, MA) were 9-10 weeks old at the time of cecal ligation and puncture (CLP) surgery. CLP surgery was performed to induce sepsis, largely as previously described (1). After a midline incision, the cecum was located and ligated with 4-0 silk suture 15 mm from the tip, and a 21-g needle was passed through the ligated cecum. The cecum was returned to the peritoneal cavity and the incision closed. During surgery, a slow-release formulation of buprenorphine (0.5 mg/kg s.c.; SR Veterinary Technologies, Windsor, CO) was administered for analgesia (1). Mice were treated with vehicle (40 ml/kg of 3% DMSO diluted with 0.9% saline) or BAM15 (5 mg/kg diluted with vehicle) i.p. at the time of surgery (0 hours) and then administered 40 ml/kg of 0.6% saline s.c. at 6 hours after CLP. In some mice, BAM15 treatment was delayed until 6 hours; these mice were given 40ml/kg 0.9% saline i.p. at the time of surgery and then administered 20ml/kg of vehicle or BAM15 (5 mg/kg) i.p. with 20 ml/kg of 0.6% saline s.c. at 6 hours after CLP. Antibiotics [14 mg/kg Primaxin (Imipenem and cilastatin), Merck, Whitehouse Station, NJ)] were given with fluids at 6 hours. In sham controls, surgery was performed as for CLP, with the cecum returned to the peritoneal cavity without ligation or puncture. For mtDNA injection model, mtDNA (400, 2000, or 8000ng) was injected into the retro-orbital vein of unoperated or postoperative mice. BAM15 (5 mg/kg) or Vehicle was administered by intraperitoneal injection. Mice were euthanized at 3 hours after mtDNA injection for sample collection. For time course of mtDNA level, blood was collected every 1 hour under anesthesia retro-orbitally from the other eye with a heparinized micro-hematocrit capillary tube (Thermo Fisher Scientific Inc, Pittsburgh, PA). The neutrophil-depleted CLP model was conducted according to a previous paper (2). CD-1 mice were injected i.p. with 500 μg αLy6G antibody (1A8; BioXCell, West Lebanon, NH) or the respective isotype control (rat IgGa, 2A3; BioXCell) 24

h and 2 h before CLP. The depletion of $Ly6G^+$ neutrophil by αLy6G antibody was confirmed by flow cytometry of mouse spleen at 18 hours after CLP or Sham.

Survival study

Mouse survival was assessed every 6 –12 hours after surgery. Antibiotic injection and fluid resuscitation were started at 6 hours after surgery by subcutaneous injection, and then repeated every 12 hours. Animals exceeding a threshold of morbidity were euthanized (3).

Tubule cell culture

Renal tubular epithelial cells (TECs) were prepared from renal cell suspension of 6 to 8-week mice following the method reported previously (4). In brief, kidneys were harvested, decapsulated, cut into small pieces with sterile instruments and digested in 30 mg/g tissue of collagenase type I (Millipore Sigma, Burlington, MA) for 30 min at 37℃. The material was pushed through a sieve of 70 µm pore (BD, Franklin Lakes, NJ), washed and diluted in 2 mL of phosphate-buffered saline (PBS). The tubule segments were separated through 31% Percoll (GE Healthcare, Uppsala, Sweden) centrifugation at 800 x g for 10 min at 4℃. The pellet was collected and washed with PBS twice at 370 x g for 5 minutes at 4℃. Isolated TECs were cultured under sterile conditions at 37℃ and 5% CO_2 in conditioned Renal Epithelial Cell Growth Medium 2 (PromoCell, Heidelberg, Germany). The TECs isolated by this method have been reported to be mainly proximal tubule epithelial cells (5).

Immunohistochemistry

After collection, tissue was immediately transferred to 10% formalin and fixed for 24 hours before paraffin embedding. Sections (4 µm) were stained with periodic acid-Schiff reagent. Semiquantitative assessment of kidney injury was performed by scoring 20 ~ 40 randomly selected, 40X objective fields on the following scale: 1) tubular damage observed in 0 –25% of the field, 2) 25–50%, 3) 50 –75%, and 4) 75–100%.

For nitrotyrosine immunohistochemistry, sections were incubated overnight at 4℃ in 1:100 dilution of rabbit polyclonal anti-nitro tyrosine antibody (ab42789; Abcam, Cambridge, MA). Slides were rinsed in PBS and developed using polyclonal goat anti-rabbit Immunoglobulins conjugated with horseradish peroxidase (HRP, Agilent Dako, Santa Clara, CA) as a secondary antibody, the manufacturer's directions. The percent positive area in each 400X-magnified field was calculated using Fiji/ ImageJ software (National Institutes of Health, Bethesda, MD). Splenic apoptosis was

assessed by activated caspase 3 as described previously (6). Immunohistochemical staining was performed with anti- caspase 3 antibody (Cell Signaling Technology, Danvers, MA) as a primary antibody and polyclonal goat anti-rabbit Immunoglobulins conjugated with HRP (Agilent Dako, Santa Clara, CA) as a secondary antibody. Neutrophils were detected using the naphthol AS-D chloroacetate esterase kit (Millipore Sigma, Burlington, MA) and was performed according to the manufacturer's specifications. Neutrophils were counted in 20X objective fields (n=5) and averaged per mouse.

Telemetry implantation and catheter insertion

For measuring blood pressure, heart rate, and body temperature measurements, mice were implanted with a radio-telemetry probe (HD-X10; Data Sciences International) as previously described (7, 8). Briefly, under isoflurane anesthesia, a neck incision was made, and the tip of catheter was inserted into the left carotid artery and secured by ligation with 6-0 silk suture. The transducer/telemetry transmitter was placed on the dorsum in a subcutaneous location. Continuous blood pressure, heart rate, and body temperature data were averaged for each minute and transmitted telemetrically. To reduce variability, we averaged consecutive 1-hour time windows starting immediately after CLP surgery.

Renal hypoxia staining in vivo

Renal hypoxia was assessed by pimonidazole immunohistochemistry. Pimonidazole (Hypoxyprobe-1 in Hypoxyprobe-1 Omni Kit; Hypoxyprobe Inc., Burlington, MA, 60 mg/kg) was administered to mice intraperitoneally at 2h before euthanasia. Kidneys were fixed with 10% formalin and processed for paraffin embedding, sectioning, peroxidase quenching and blocking as described previously (9). The sections were sequentially incubated with rabbit anti-pimonidazole antibody (PAb2627; Hypoxyprobe Inc., Burlington, MA), and subsequently polyclonal goat anti-rabbit immunoglobulins conjugated with HRP (Agilent Dako, Santa Clara, CA), following the manufacturer instructions.

Renal hypoxia was evaluated by semiquantitative measurements of pimonidazole staining on tubules of the cortex and the outer stripe of the OSOM. The degree of pimonidazole staining was estimated at 400X magnification using more than five randomly selected fields for each animal, and scored according to the intensity and the extent of positive cells: 0, no staining; 1, minimal, 2; moderate, 3; severe staining.

Clinical chemistry markers and cytokines

An autoanalyzer (Hitachi 917; Boehringer Mannheim, Indianapolis, IN) was used to measure serum BUN, lactate dehydrogenase, alkaline phosphatase, aspartate transaminase. Serum creatinine was measured by HPLC (11). Serum IL-6, IL-10, IL-17, and TNFα were measured by ELISA (R & D Systems, Minneapolis, MN).

mtDNA isolation

For *in vivo* and *in vitro* studies with mtDNA, mice were subjected to either sham or CLP surgery, then 18 hours later, livers were removed, and liver mitochondria were isolated with Mitochondria Isolation Kit for Tissue (Abcam, Cambridge, MA). MtDNA was then extracted from the mitochondria using DNeasy Blood & Tissue kit (Qiagen, Germantown, MD). The amount of mtDNA was measured by Qubit 1XdsDNA HS Assay Kit with Qubit 4 Fluorometer (Thermo Fisher Scientific, Waltham, MA).

Isolation of Bacterial DNA

Blood was collected under anesthesia and sterile conditions at 18 hours after CLP surgery and plated on 5% sheep blood agar (BD, Franklin Lakes, NJ) and incubated at 37°C for 24 hours. Single colonies were collected and incubated in 3ml LB broth (Quality Biological, Inc., Gaithersburg, MD) at 37°C for 24 hours with shaking at 200 rpm. Bacterial DNA was extracted from the bacteria-cultured LB broth by using QIAamp DNA Microbiome kit (Qiagen, Germantown, MD) according to the manufacturer's instruction. The appropriate concentration was prepared with measuring by Qubit 1XdsDNA HS Assay Kit with Qubit 4 Fluorometer (Thermo Fisher Scientific, Waltham, MA).

Treatment with potassium superoxide

Potassium superoxide (KO_2, Millipore Sigma, Burlington, MA) was dissolved in DMSO and applied to mPPTCs ($5x10^5$) at final concentration of 0 (without KO_2), 1, 10, and 100μM. The supernatant of medium of mPPTCs was collected at 0 and 3 hours after incubation at 37℃ and 5% CO_2.

qPCR of mtDNA

DNA were extracted with the QIAamp DNA Mini and Blood Mini kit (Qiagen, Germantown, MD) from plasma, urine, and supernatant of medium after 2-step centrifugation (1,000 x g for 10 min, followed by 16,000 x g for 10 min). Taqman primers (forward: 5'-TCATGTCGGACGAGGCTTAT-3', reverse: 5'-CTCATGGAAGGACGTAGCCT-3') and a probe (5'-ACTGTTCGCAGTCATAGCCACAGCA-3') with a unique 5′ fluorescent reporter dye (FAM) and

3 fluorescent quencher dye (TAMRA) were designed using mouse mitochondrial genome sequences and synthesized by Genscript (Piscataway, NJ). For absolute quantification using qPCR, a mitochondrial DNA standard was prepared by PCR of liver mitochondrial DNA with these primers, purified using a QIAquick PCR Purification Kit (Qiagen, Germantown, MD), and serially diluted. The concentration of the mitochondrial DNA standard was measured by Qubit 1XdsDNA HS Assay Kit with Qubit 4 Fluorometer (Thermo Fisher Scientific, Waltham, MA). With the standard curves of the targeted mtDNA, plasma and urinary mtDNA in samples was quantified by the following thermal profile used with QuantStudio™ 6 Flex System (Applied Biosystems, Carlsbad, CA, consisting of an initial cycle of 2 minutes at 95 °C, followed by 40 cycles of 5 seconds at 95 °C and 10 seconds at 54 °C. The plasma concentration of mtDNA is expressed as copies per microliter; urine mtDNA is expressed as copies per gram creatinine, as measured by Creatinine Companion (Ethos Biosciences, Newtown Square, PA). The previous method (10) using 3 primers mouse Nd1, CytB, COX3 with SYBR green dye were reproduced for confirming the compatibility of this mtDNA quantification method.

Measuring mitochondrial respiratory function

A Cell Mito Stress Test (Agilent, Santa Clara, CA) was performed following the manufacturer's protocol on a Seahorse XFe96 extracellular flux analyzer (Agilent). TECs were plated on a sterile XFe96 well plate in duplicate at 2×10^4 total cells per well and serially stimulated in the following sequence: (A) 1μM oligomycin; (B) 0-100 μM carbonyl cyanide-4-(trifluoromethoxy) phenylhydrazone (FCCP) or BAM15; (C) 2 μM rotenone/antimycin A; (D) 4μg/ml Hoechst 33342. The plate was returned to the Cytation 5 (Bioteck, Winooski, VT) for a fluorescence scan and cell count. Normalization, calculation, and visualization of respiratory parameters including basal oxygen consumption rate (OCR) and maximum respiration were performed by Wave software (Agilent, Santa Clara, CA).

Live cell imaging of mtROS

mPPTCs ($5x10^4$) were plated onto a chambered cover glass and incubated in phenol-red-free low glucose (5.5mM) medium for one day and stained with 200nM MitoSOX-Red and 2 μg/ml Hoechst 33343 (Thermo Fisher Scientific, Waltham, MA) for 30 min at 37℃. PTECs were washed with Hanks' balanced salt solution (HBSS) with Ca and Mg and were incubated with ProLong Live Antifade Reagent (Thermo Fisher Scientific, Waltham, MA). Serum was collected from mice at 18 hours after CLP or sham surgery and was filtered through a 0.22 μm pore filter. Renal Epithelial Cell Growth Medium 2 (PromoCell, Heidelberg, Germany) was supplemented with a) 10% serum

from mice subjected to either CLP or sham surgery (collected 18 hours post-surgery) and b) 10 or 20 μM BAM15 or the equivalent volume of vehicle. PTECs were incubated in these media. Images of cultured cells were acquired by confocal microscopy with incubation system (Zeiss LSM780, Zeiss, Oberkochen, German) at 0, 6, 12 and 24 hours after serum addition. Isolated mtDNA (1ng) from liver of Sham or CLP mice also were incubated with mPPTCs. The images were acquired at 3 hours after adding mtDNA. The fluorescence of MitoSOX-Red and Hoechst 33343 were imaged at 488 nm and 405 nm excitation and using 562nm-666nm and 413mm-490nm emission ranges. All imaging parameters remained the same during data acquisition, and images were processed identically and exported using Zen Zeiss software. The relative fluorescence units of individual cells were quantified using Fiji/ ImageJ software. Corrected total cell fluorescence units were determined using the following formula: integrated density of selected cell − (area of selected cell × mean integrated density of background readings).

Bacterial counts

Blood specimens and peritoneal exudate, obtained after injection of 0.9% saline (2 ml) into the peritoneal cavity, were collected under sterile conditions at 18 hours after CLP or Sham surgery. Serial dilutions were plated on 5% sheep blood agar (BD, Franklin Lakes, NJ) and incubated at 37°C. The number of colonies was counted after culture for 24 hours at 37℃.

Cecal material was collected from healthy CD1-mice and diluted with 10ml of PBS as a bacterial suspension of CLP. Fifty μl of bacterial suspension, treated with 0, 1, 5, 10, 20 or 50 μM BAM15, was applied to BBL trypticase soy agar with 5% sheep blood (TSAⅡ) (BD, Franklin Lakes, NJ). The number of colonies was counted after culturing for 24 hours at 37℃.

Western-blotting analysis

Total protein was extracted from whole kidney based using a bead-based Precellys 24 tissue homogenizer (Bertin Technologies, Montigny-le-Bretonneux, France) with T-PER Tissue Protein Extraction Reagent (Thermo Fisher Scientific, Waltham, MA) treated with Halt Phosphatase Inhibitor Cocktail (Thermo Fisher Scientific, Waltham, MA) and Complete Mini Protease Inhibitor Cocktail (Millipore Sigma, Burlington, MA). Lysate protein concentrations were measured by BCA assay and adjusted to be the same. Protein lysates were denatured by mixing with an equal volume of 2X Laemmli sample buffer (with 0.2 M DTT) and heated for 10 min at 95°. Samples were resolved by SDS-PAGE and transferred to nitrocellulose membranes using an iBlot dry blotting system (Invitrogen, Carlsbad, CA). Membranes were blocked for 30 min with blocking buffer for fluorescent Western blotting (Rockland Immunochemicals, Inc., Pottstown, PA) and

incubated with the primary antibody overnight at 4℃. Membranes were washed and incubated with secondary antibody for 1 hour at room temperature. Primary antibodies included anti-AMPKα mouse antibody (1:500, #2793), Phospho-AMPKα rabbit antibody (1:500, #2535), anti-Sirt1 rabbit antibody (1:1000, #9475, all from Cell Signaling Technology, Danvers, MA) anti-PGC1α rabbit antibody (1:1000, NBP1-04676, Novus Biologicals, Centennial, CO) anti-TFAM rabbit antibody (1:1000, SAB 1401383, Sigma-Aldrich, Saint Louis, MO), anti-β-actin mouse antibody as a control (1:5000, #3700, Cell Signaling Technology, Danvers, MA). The primary antibody for quantification of mitochondrial contents was MitoBiogenesis™ Western Blot Cocktail (1:250, ab123545, Abcam, Cambridge, MA) containing mouse SDHA monoclonal antibody, mouse COX-1 monoclonal antibody and mouse β-actin monoclonal antibody. Labeled secondary antibodies included IRDye 680LT Goat anti-rabbit IgG secondary antibody (1:4000) and IRDye 800CW donkey anti-mouse IgG secondary antibody (1:3000, both from LI-COR Biosciences, Lincoln, NE). Western blot membranes were imaged by two-color Western blot detection with infrared fluorescence with a LI-COR Odyssey infrared scanner (LI-COR Biosciences, Lincoln, NE). Band intensities were analyzed using Odyssey software.

Quantitation of NAD^+/NADH in kidney

Fresh kidneys were harvested from mice and 25 mg of the cortex was used for assay. Kidneys were homogenized by a bead-based Precellys 24 tissue homogenizer (Bertin Technologies, Montigny-le-Bretonneux, France). NAD^+ and NADH in kidney lysate were measured following the procedure of BioAssay Systems' EnzyChrom NAD^+/NADH assay kit (BioAssay Systems, Hayward, CA). The optical density (OD) values for time “zero” and OD15 after a 15-min incubation were obtained at 565 nm.

Flow cytometry analysis

Spleens were harvested from mice and dissociated with the rough side of frosted glass slides. Cell suspensions were collected in MACS buffer (Miltenyi Biotech, Auburn, CA) by passage through 40 µM Nylon filter (BD Falcon, Bedford, MA) followed by red blood cell lysis using 3 ml/spleen of ACK lysing buffer (Quality Biological, Gaithersburg, MD). 5 x 10^6 cells per tube were prepared for flow cytometry. Cells were incubated in Fc Block (1:200, #553142, BD Biosciences, Sparks, MD) for 10 min at 4 ℃. Surface staining was performed in the dark for 15 min at 4 ℃. Cell viability was assessed with Live/Dead™ Yellow Dead cell staining kit (1:1000, L34959, Invitrogen, Carlsbad, CA) according to the manufacturer’s instructions. A comprehensive list of

surface markers includes: BUV395 Rat Anti-Mouse CD45 (Clone 30-F11, 1:100, #56429, BD Biosciences), BV711 Hamster Anti-Mouse CD3e (Clone 145-2C11, 1:200, #563123, BD Biosciences) for T cells, PerCP-Cyanine 5.5 Anti-Mouse CD19 (Clone 1D3, 1:800, #65-0193, Tonbo™ A Cytek® Brand, San Diego, CA) for B cells, PE/Cyanine7 anti-mouse NK-1.1 (Clone PK136, 1:100, #108714, BioLegend, San Diego, CA) for NK-T cells, APC/Cyanine7 anti-mouse/human CD11b (Clone M1/70, #101226, 1;1500, BioLegend), APC anti-mouse Ly6C (Colone HK1.4, #128015, 1:200, BioLegend), BUV563 Rat Anti-Mouse Ly6G (Clone 1A8, 1:100, #612921, BD Biosciences), for Myeloid cells, Alexa Fluo® 488 anti-mouse CD11c (Clone N418, 1:200, #117313, BioLegend), Alexa Fluor® 700 anti-mouse/human CD45R/B220 (Clone RA3-6B2, 1:200, #103232, BioLegend) for plasmacytoid dendritic cells. Gating strategy for immunophenotyping in myeloid cells was based on the method of Rose et al. (12). Absolute cell number in a spleen was counted by using CountBright Absolute Counting beads (#C36950, Invitrogen) and divided by spleen weight (mg). Apoptotic cells were assessed with NucView® 405 Caspase-3 Substrate (1:500, #76204-794, Biotium, Fremont, CA). Cells were acquired on BD FACSymphony™ (BD Life Sciences, San Jose, CA). A titration test was performed for all antibodies and reagents in advance. Fluorescence minus one (FMO) controls were used for all gating analyses to distinguish positively from negatively staining cell populations. Compensation was performed using single color controls prepared from UltraComp eBeads Plus compensation beads (Thermo Fisher Scientific, Waltham, MA). Compensation matrices were calculated and applied using FlowJo software (Tree Star, Ashland, OR).

Isolation of Neutrophils

Neutrophils were isolated by Ly6G-based positive immunomagnetic selection from mouse splenocytes according to established methods (13). Spleen was obtained from mice following perfusion under anesthesia, and gently ground in RPMI supplemented with 10% FBS, 1% penicillin/streptomycin, and 60 U/ml DNase I (Millipore Sigma, Burlington, MA). The ground spleen was incubated for 30 min at 37 ℃ followed by passing it through a 100 μm cell strainer. After centrifugation and washing, splenocytes were resuspended in MACS buffer. Anti-Ly-6G MicroBeads Ultrapure were added and incubated according to the manufacturer's instruction (Miltenyi Biotec, Bergisch, Gladbach, Germany). Subsequently, Ly6G+ neutrophils are collected by separation over a LS Column placed in the magnetic field of a MACS Separator. Isolated Ly6G+ neutrophils were suspended in RPMI supplemented with 10% FBS, 1% penicillin/streptomycin.

Neutrophil in-vitro experiment

Ly6G+ neutrophils ($5x10^5$) were plated and stimulated for 3 hours at 37 °C and 5% CO_2 with 100 nM PMA or 25 μg/ml mtDNA (originating from mitochondria of CLP liver, see above) using a modified method of previous papers (14, 15). As treatment for these stimulated neutrophils, BAM15 (20 μM) or Vehicle were also applied with stimulators. Supernatant of medium of neutrophils were collected at 3 hours after incubation and processed for measurement of mtDNA level by qPCR (see above). To assess the extent of mtDNA amount released from neutrophils by applying mtDNA, we calculated mtDNA increase ratio as [(mtDNA amount in medium – applied mtDNA amount)/applied mtDNA amount] after quantification by qPCR.

References

1. Miyaji T, Hu X, Yuen PS, Muramatsu Y, Iyer S, Hewitt SM, et al. Ethyl pyruvate decreases sepsis-induced acute renal failure and multiple organ damage in aged mice. Kidney Int. 2003;64(5):1620-31.

2. Ocuin LM, Bamboat ZM, Balachandran VP, Cavnar MJ, Obaid H, Plitas G, et al. Neutrophil IL-10 suppresses peritoneal inflammatory monocytes during polymicrobial sepsis. J Leukoc Biol. 2011;89(3):423-32.

3. Zafrani L, Gerotziafas G, Byrnes C, Hu X, Perez J, Levi C, et al. Calpastatin controls polymicrobial sepsis by limiting procoagulant microparticle release. Am J Respir Crit Care Med. 2012;185(7):744-55.

4. Iwakura T, Zhao Z, Marschner JA, Devarapu SK, Yasuda H, and Anders HJ. Dipeptidyl peptidase-4 inhibitor teneligliptin accelerates recovery from cisplatin-induced acute kidney injury by attenuating inflammation and promoting tubular regeneration. Nephrol Dial Transplant. 2019;34(10):1669-80.

5. Terryn S, Jouret F, Vandenabeele F, Smolders I, Moreels M, Devuyst O, et al. A primary culture of mouse proximal tubular cells, established on collagen-coated membranes. Am J Physiol Renal Physiol. 2007;293(2):F476-85.

6. Dear JW, Yasuda H, Hu X, Hieny S, Yuen PS, Hewitt SM, et al. Sepsis-induced organ failure is mediated by different pathways in the kidney and liver: acute renal failure is dependent on MyD88 but not renal cell apoptosis. Kidney Int. 2006;69(5):832-6.

7. Doi K, Hu X, Yuen PS, Leelahavanichkul A, Yasuda H, Kim SM, et al. AP214, an analogue of alpha-melanocyte-stimulating hormone, ameliorates sepsis-induced acute kidney injury and mortality. Kidney Int. 2008;73(11):1266-74.

8. Yamashita T, Street JM, Halasa BC, Naito Y, Tsuji T, Tsuji N, et al. The effect of continuous intravenous norepinephrine infusion on systemic hemodynamics in a telemetrically-monitored mouse model of sepsis. PLoS One. 2022;17(8):e0271667.

9. Yasuda H, Yuen PS, Hu X, Zhou H, and Star RA. Simvastatin improves sepsis-induced mortality and acute kidney injury via renal vascular effects. Kidney Int. 2006;69(9):1535-42.

10. Tsuji N, Tsuji T, Ohashi N, Kato A, Fujigaki Y, and Yasuda H. Role of Mitochondrial DNA in Septic AKI via Toll-Like Receptor 9. J Am Soc Nephrol. 2016;27(7):2009-20.

11. Yuen PS, Dunn SR, Miyaji T, Yasuda H, Sharma K, and Star RA. A simplified method for HPLC determination of creatinine in mouse serum. Am J Physiol Renal Physiol. 2004;286(6):F1116-9.

12. Rose S, Misharin A, and Perlman H. A novel Ly6C/Ly6G-based strategy to analyze the mouse splenic myeloid compartment. Cytometry A. 2012;81(4):343-50.

13. Swamydas M, Luo Y, Dorf ME, and Lionakis MS. Isolation of Mouse Neutrophils. Curr Protoc Immunol. 2015;110:3 20 1-3 15.

14. Mallavia B, Liu F, Lefrancais E, Cleary SJ, Kwaan N, Tian JJ, et al. Mitochondrial DNA Stimulates TLR9-Dependent Neutrophil Extracellular Trap Formation in Primary Graft Dysfunction. Am J Respir Cell Mol Biol. 2020;62(3):364-72.

15. Remijsen Q, Vanden Berghe T, Wirawan E, Asselbergh B, Parthoens E, De Rycke R, et al. Neutrophil extracellular trap cell death requires both autophagy and superoxide generation. Cell Res. 2011;21(2):290-304.

Supplemental figure 1

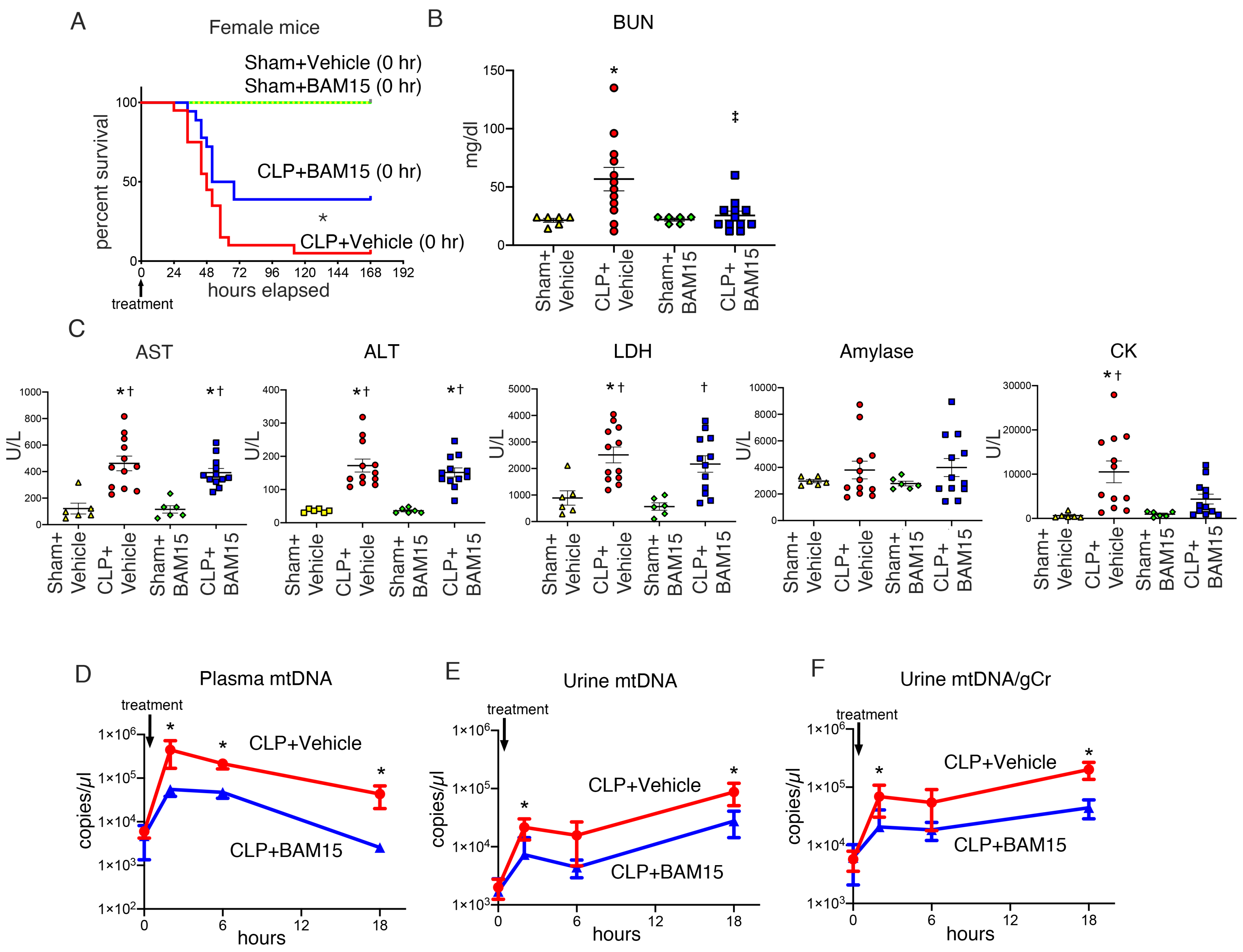

Supplemental Figure 1. BAM15 treatment improves mortality and AKI in septic female mice. (**A**) Kaplan-Meier curves of Sham or CLP female mice treated with vehicle (at 0 hours) and Sham or CLP treated with BAM15 (5 mg/kg, at 0 hours) for 7 days. Sham + Vehicle or BAM15: n=4 each, CLP+ Vehicle or BAM15: n=20 each. Log-rank test. *$p<0.05$, CLP + Vehicle vs other groups. (B-C) BUN (**B**), AST, ALT, LDH, Amylase, CK (**C**) by biochemical examination at 18hrs after Sham or CLP female mice treated with vehicle (at 0 hours) or BAM15 (5 mg/kg, at 0 hours). All bars show mean ± SEM of each group (Sham + Vehicle: n=6, CLP + Vehicle: n=12, Sham + BAM15: n=6, CLP + BAM15: n=12). Dunn's multiple comparisons test following Kruskal Wallis test. *: vs Sham + Vehicle, $p<0.05$. †: vs Sham + BAM15, $p<0.05$. ‡: vs CLP + Vehicle, $p<0.05$. (D-F) Time course of plasma mtDNA level (**D**) and urine mtDNA (**E**) and urine mtDNA adjusted to creatinine excretion (**F**) at 18 hours after CLP (n=12 each) mice treated with vehicle (at 0 hours) or BAM15 (5 mg/kg, at 0 hours). The data represent mean ± SEM. Analysis between groups at each time point was performed with Sidak's multiple comparisons test following mixed-effects analysis. *: $p<0.05$.

Supplemental figure 2

A

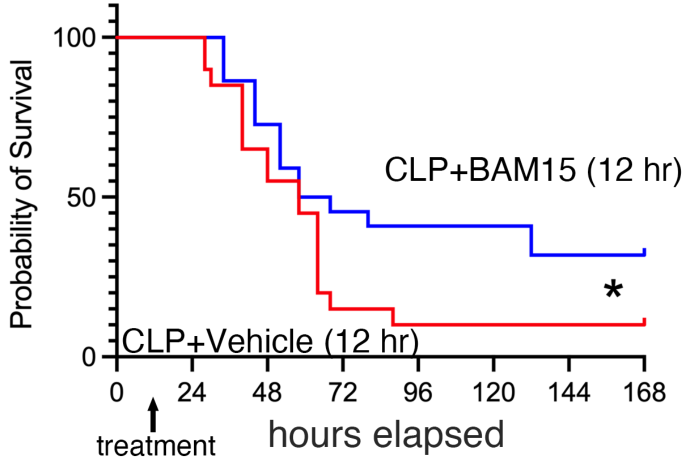

;upplemental Figure 2. BAM15 treatment improves nortality in septic mice with delayed (at 12 hours) ıdministration. (**A**) Kaplan-Meier curves of CLP male nice treated with vehicle (at 12 hours) and CLP treated vith BAM15 (5 mg/kg, at 12 hours) for 7 days. n=20 (CLP+ /ehicle), n=22 (CLP + BAM15). Log-rank test. *$p<0.05$.

Supplemental figure 3

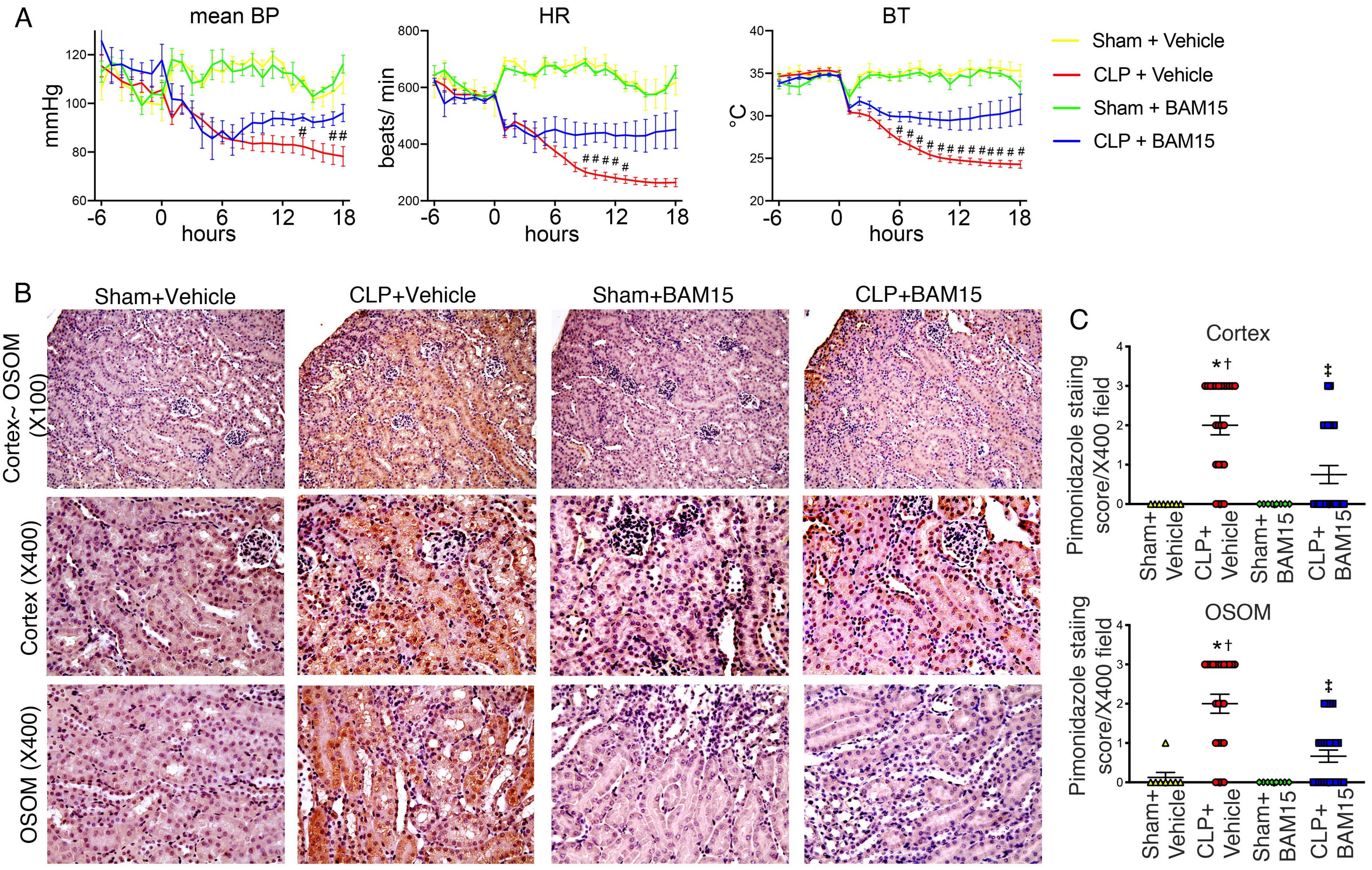

Supplemental Figure 3 (**A**) Continuous mean blood pressure (mBP), heart rate (HR), and body temperature (BT) before and after Sham or CLP surgery with vehicle (at 0 hours) or BAM15 (5 mg/kg, at 0 hours). Data were averaged across consecutive 1-hour windows. The bars show mean of ± SEM of 1-hour window-data of all mice in each group. CLP + Vehicle vs CLP + BAM15. Sham + Vehicle: n=3, CLP + Vehicle: n=9, Sham + BAM15: n=3, CLP + BAM15: n=8. Tukey's multiple comparisons test following two-way ANOVA analysis. #: $p<0.05$, CLP+ Vehicle vs CLP + BAM15. (B-C) Pimonidazole staining of cortex and outer stripe of the outer medulla (OSOM) in kidney at 18 hours after Sham or CLP mice treated with vehicle (at 0 hours) or BAM15 (5 mg/kg, at 0 hours). (**B**) Representative images of cortex and OSOM. Original magnification, upper: 100X, middle and lower: 400X. (**C**) Tubular hypoxia score in cortex and OSOM of kidney at 18 hours after Sham or CLP treated with vehicle (at 0 hours) or BAM15 (5 mg/kg, at 0 hours). Each group has 4 mice and total 24 fields for CLP groups and 8 fields for Sham groups. All graphs represent mean ± SEM. Dunn's multiple comparisons test following Kruskal Wallis test. *: vs Sham + Vehicle, $p<0.05$. †: vs Sham + BAM15, $p<0.05$. ‡: vs CLP + Vehicle, $p<0.05$.

Supplemental figure 4

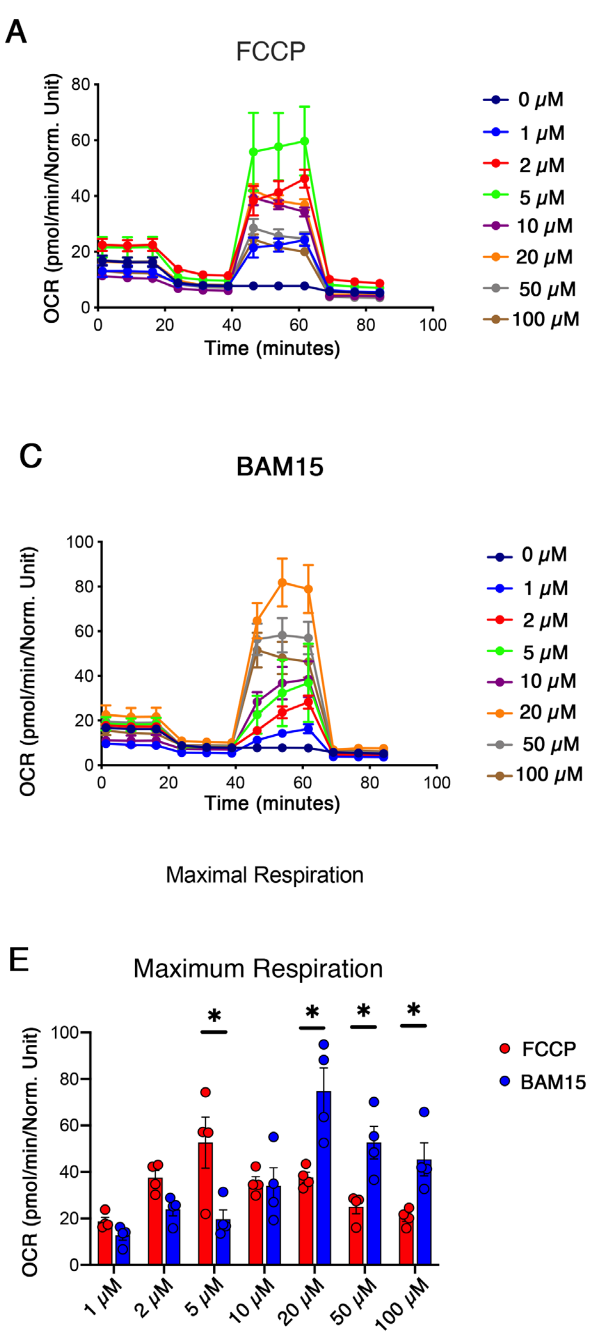

Supplemental Figure 4. Biological activity of BAM15 on mitochondrial respiratory chain as an uncoupler. (A-D) OCR and Maximum respiration of FCCP (**A, B**) and BAM15 (**C, D**) with 4 biological replicates per concentration (0, 1, 2, 5, 10, 20, 50, and 100μM) and the concentration dependence on maximum respiration (**E**). Tukey's multiple comparisons test following one-way ANOVA test (B, D) and Sidak's multiple comparisons test following two-way ANOVA test (E). *: $p<0.05$.

Supplemental figure 5

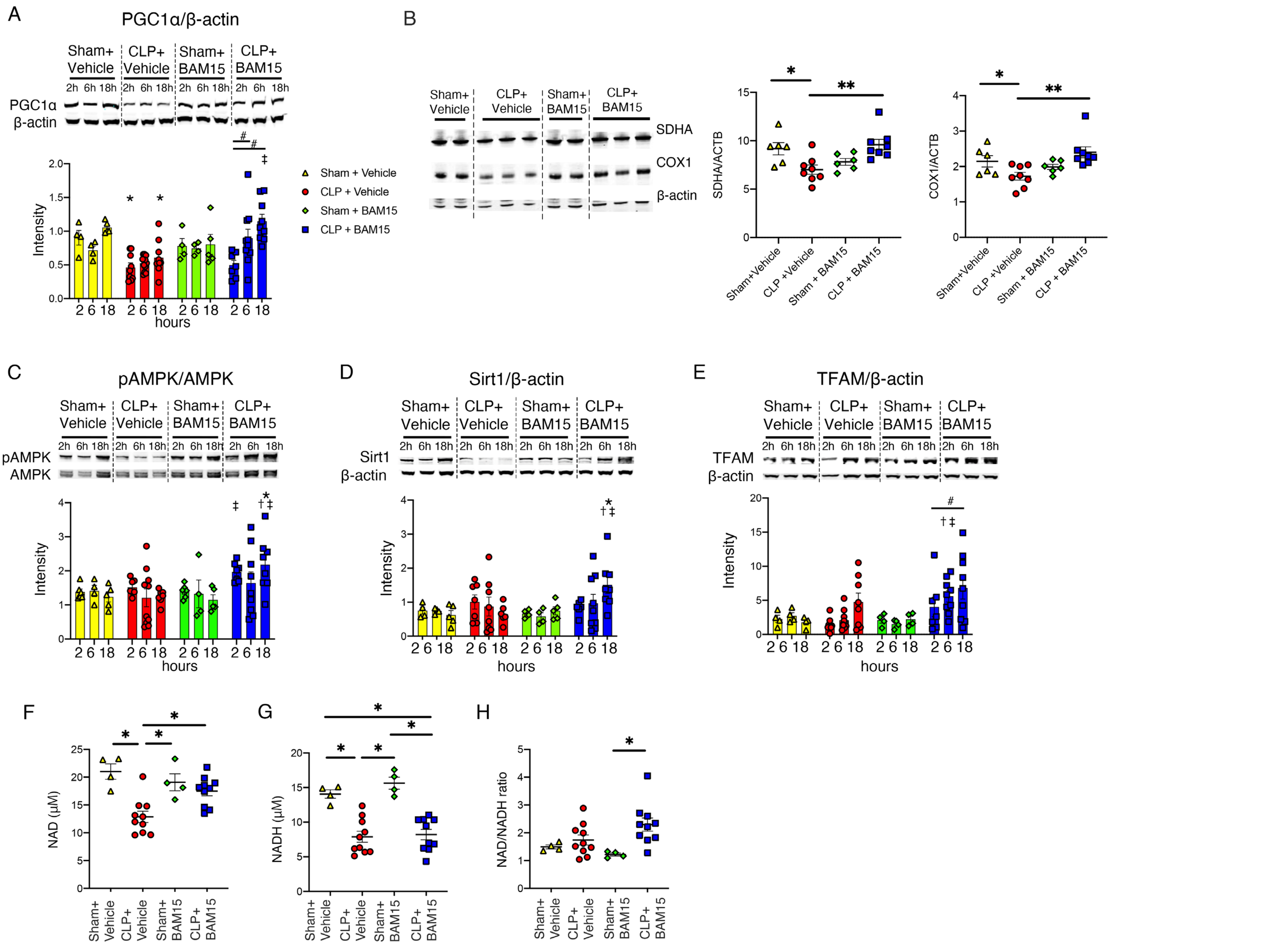

Supplemental Figure 5. BAM15 recovers mitochondrial biogenesis in septic kidney via PGC1-α with increasing its regulator, AMPK and Sirt1 and increasing TFAM. (A-E) Representative blot image and fluorescent intensity of western-blotting of PGC1-α (**A**), mitochondrial protein (SDHA, COX1, only at 18 hours) (**B**), Sirt1 (**C**), phosphorylated AMPK/AMPK, (**D**) and TFAM (**E**) in whole kidney at 2, 6 and 18 hours after Sham or CLP mice treated with vehicle (at 0 hours) or BAM15 (5 mg/kg, at 0hrs). β-actin is the internal control. n=4-11 mice per each group. All bars represent mean ± SEM. Tukey's multiple comparisons test following two-way ANOVA test. *: vs Sham + Vehicle, $p<0.05$. †: vs Sham + BAM15, $p<0.05$. ‡: vs CLP + Vehicle, $p<0.05$. #: comparison between time points, $p<0.05$. (F-H) Concentration of NAD^+ (**F**), NADH (**G**), NAD^+/NADH ratio (**H**) in kidney cortex at 18 hours after Sham or CLP mice treated with vehicle (at 0 hours) or BAM15 (5 mg/kg, at 0 hours). n=4 for each sham group, n=10 for each CLP group. All graphs represent mean ± SEM. Tukey's multiple comparisons test following one-way ANOVA test. *: $p<0.05$.

Supplemental figure 6

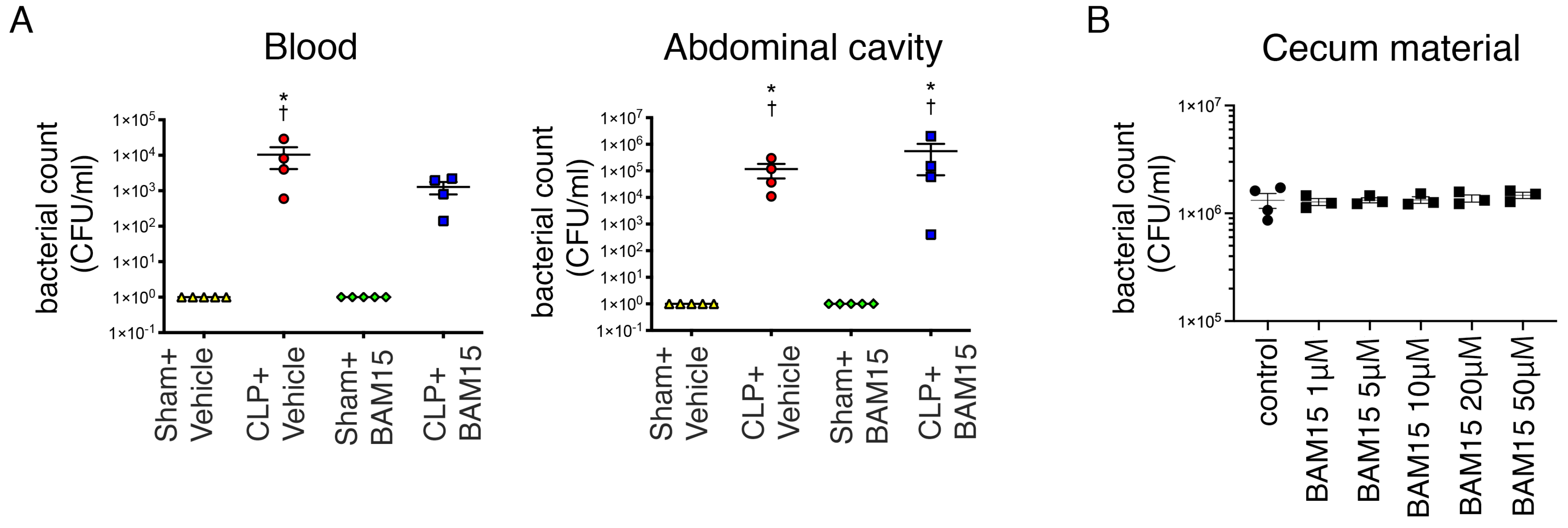

Supplemental Figure 6. BAM15 does not kill bacteria. (**A**) Bacterial count in blood and fluid of abdominal cavity at 18 hours after Sham (n=5 each) or CLP (n=4 each) mice treated with vehicle (at 0 hours) or BAM15 (5 mg/kg, at 0 hours). (**B**) Bacterial count in suspension of cecum material treated with 0, 1, 5, 10, 20, and 50 μM BAM15 (n=3~4). All graphs represent mean ± SEM. Tukey's multiple comparisons test following one-way ANOVA test. * vs Sham + Vehicle, $p<0.05$. † vs Sham + BAM15, $p<0.05$. ‡ vs CLP + Vehicle, $p<0.05$.

Supplemental figure 7

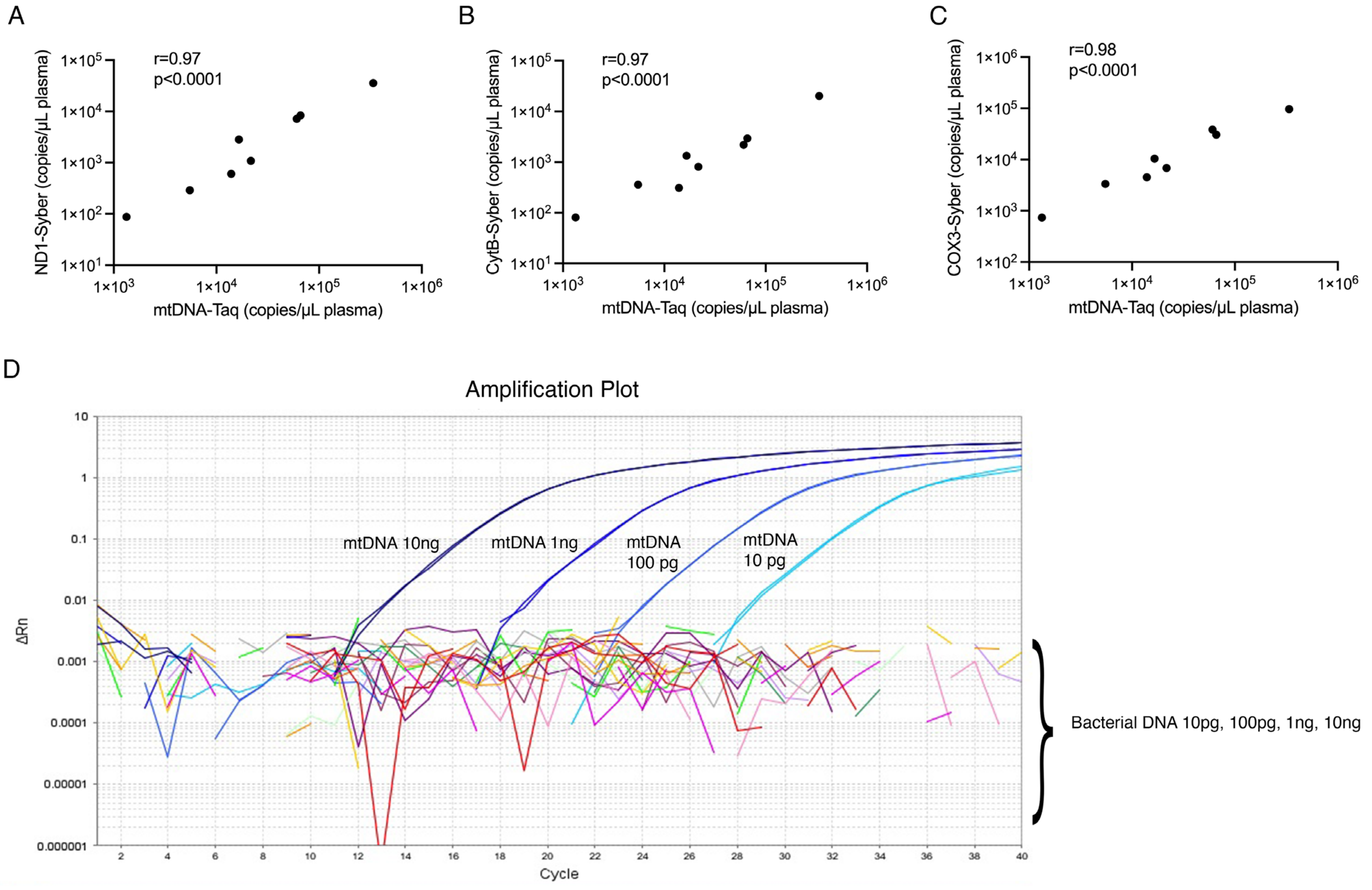

Supplemental Figure 7. Verification of mtDNA quantification in this study. (A-C) Correlation between mtDNA level measured by our new quantification method (mtDNA-Taq) and the previous methods: ND1-Sybr Green (**A**), CytB-Sybr Green (**B**), and COX3-Sybr Green (**C**). mtDNA were quantified by each qPCR method by using 8 plasma samples. r value is Pearson's correlation coefficient, p<0.00001. (**D**) Amplification plot of mtDNA and bacterial DNA (10 pg, 100 pg, 1ng, or 10ng) by using our new quantification method. Bacterial DNA (n=3) was extracted from blood agar plates cultured with blood from three CLP mice, respectively. mtDNA was extracted from liver mitochondria of naïve mice under sterile conditions. No amplification of mtDNA was detected in any bacterial DNA.

Supplemental figure 8

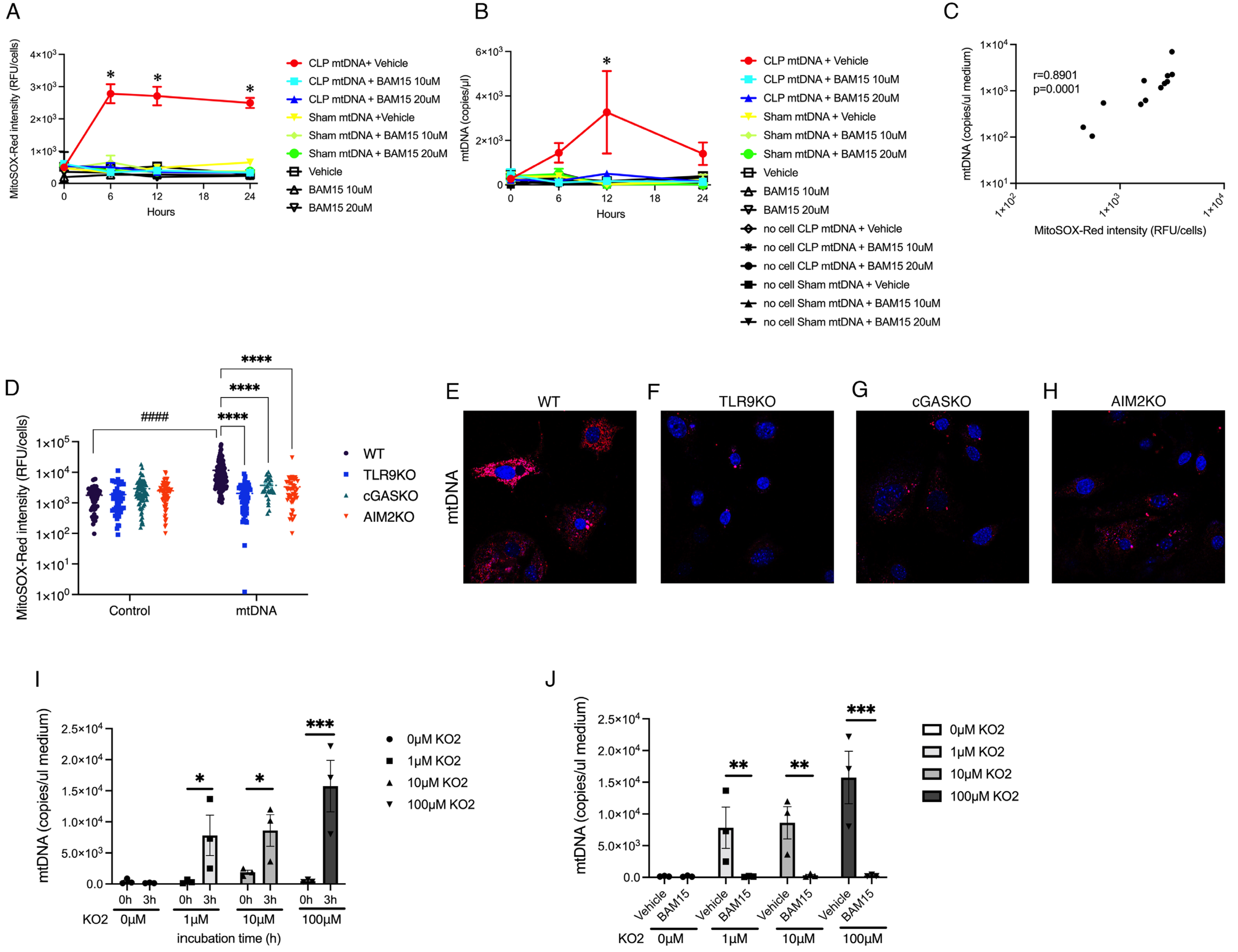

Supplemental Figure 8. Generation of mtROS in kidney tubule cells by sepsis-derived mtDNA. (A-C) Serial live cell imaging of mtROS in mPPTCs incubated with Vehicle/BAM15 (10μM or 20μM) and mtDNA (1 ng) purified from liver of CLP or Sham mice. (**A**) Time course of fluorescence intensity of MitoSOX-Red in a well serially measured. The graph represents mean ± SEM. Tukey's multiple comparisons test following two-way ANOVA test. 6-10 randomized images (400X) on 3 biological replicates per each condition. *p<0.05: CLP + mtDNA vs every other group. Tukey's multiple comparisons test following two-way ANOVA test. (**B**) Time course of extracellular mtDNA level of the supernatant of (A). The graph represents mean ± SEM. *p<0.05: CLP + mtDNA vs every other group. Tukey's multiple comparisons test following two-way ANOVA test. (**C**) Correlation between extracellular mtDNA level and the corresponding MitoSOX-Red intensity in the mPPTCs incubated with mtDNA (1ng) treated with vehicle. n=12 from 0, 6, 12 and 24h on 3 biological replicates. r value is Pearson's correlation coefficient, *p<0.05. (**D**) MitoSOX-Red intensity in a well at 6 hours after treatment with mtDNA (1ng) in mPPTCs purified from WT, TLR9KO, cGASKO, and AIM2 KO mice. The graph represents mean ± SEM. 6-10 fields (400X) on 2 biological replicates. ****p<0.00001: vs cell type, $^{\#\#\#\#\#}$p<0.0001: control vs mtDNA. Tukey's multiple comparisons test following two-way ANOVA test. (**E-H**) Representative MitoSOX-Red images of (D). Red: MitoSOX-Red, Blue: Hoechst 33342. Original magnification, 400X. (**I**) Extracellular mtDNA level of the supernatant at 3 hours of mPPTCs treated with graded concentration of superoxide (KO_2: 0, 1, 10, 100 mM). The graph represents mean ± SEM. n=3 each. *p<0.05, ***p<0.001. unpaired *t* test. (**J**) Comparison of extracellular mtDNA level of the supernatant of superoxide-treated mPPTCs with vehicle and BAM15 (20 mM). The graph represents mean ± SEM. **p<0.01, ***p<0.001. unpaired *t* test.

Supplemental figure 9

A

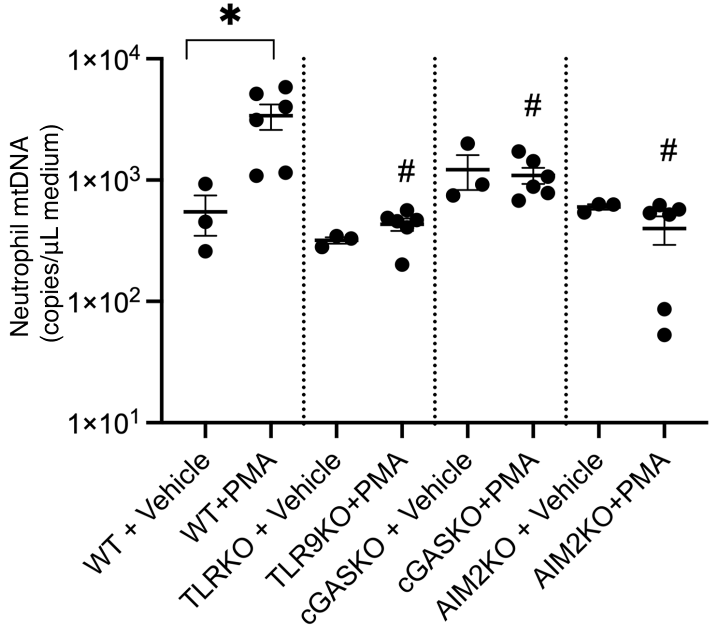

B

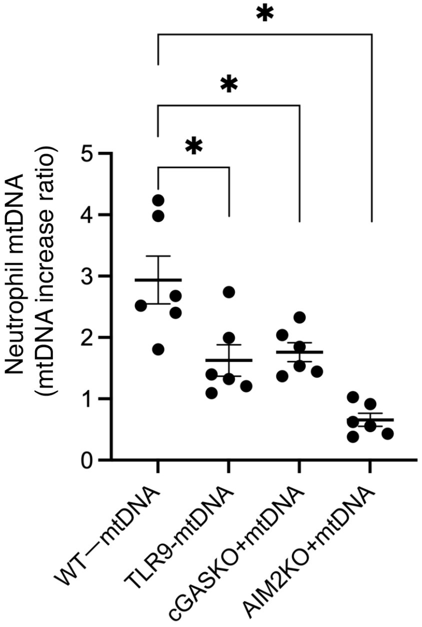

Supplemental Figure 9. mtDNA is released from neutrophils via TLR9, cGAS, AIM2 pathway. (**A**) Extracellular mtDNA levels in medium of Ly6G⁺ neutrophils of TLR9, cGAS, AIM2 knockout mice at 3 hours after treatment with PMA with or without BAM15. n=6 each for PMA treatment groups, n=3 each for vehicle treatment groups. The graph represent mean ± SEM. *p<0.05: vehicle vs PMA treatment for wild-type or each knockout cell type, unpaired *t* test. #p<0.05: cells from WT vs each KO, Sidak's multiple comparisons test following one-way ANOVA test. (**B**) Extracellular mtDNA increase ratio in medium of Ly6G⁺ neutrophils of TLR9, cGAS, AIM2 knockout mice at 3 hours after treatment with mtDNA (25μg/ml). mtDNA increase ratio was calculated by [(mtDNA amount in medium – applied mtDNA amount)/applied mtDNA amount]. n=6 each. The graph represents mean ± SEM. Sidak's multiple comparisons test following one-way ANOVA test. *p<0.05: WT + mtDNA vs each KO + mtDNA.
